# *Desulfatiglans anilini* Initiates Degradation of Aniline With the Production of Phenylphosphoamidate and 4-Aminobenzoate as Intermediates Through Synthases and Carboxylases From Different Gene Clusters

**DOI:** 10.3389/fmicb.2020.02064

**Published:** 2020-09-04

**Authors:** Xiaoman Xie, Dieter Spiteller, Thomas Huhn, Bernhard Schink, Nicolai Müller

**Affiliations:** ^1^Department of Biology, Universität Konstanz, Konstanz, Germany; ^2^Konstanz Research School Chemical Biology, Konstanz, Germany; ^3^Department of Chemistry, Universität Konstanz, Konstanz, Germany

**Keywords:** aniline, *Desulfatiglans anilini*, phenylphosphate carboxylase, phenylphosphate synthase, aromatic degradation, sulfate reduction

## Abstract

The anaerobic degradation of aniline was studied in the sulfate-reducing bacterium *Desulfatiglans anilini*. Our aim was to identify the genes and their proteins that are required for the initial activation of aniline as well as to characterize intermediates of this reaction. Aniline-induced genes were revealed by comparison of the proteomes of *D. anilini* grown with different substrates (aniline, 4-aminobenzoate, phenol, and benzoate). Most genes encoding proteins that were highly abundant in aniline- or 4-aminobenzoate-grown *D. anilini* cells but not in phenol- or benzoate-grown cells were located in the putative gene clusters *ani* (aniline degradation), *hcr* (4-hydroxybenzoyl-CoA reductase) and *phe* (phenol degradation). Of these putative gene clusters, only the *phe* gene cluster has been studied previously. Based on the differential proteome analysis, four candidate genes coding for kinase subunits and carboxylase subunits were suspected to be responsible for the initial conversion of aniline to 4-aminobenzoate. These genes were cloned and overproduced in *E. coli*. The recombinant proteins were obtained in inclusion bodies but could be refolded successfully. Two subunits of phenylphosphoamidate synthase and two carboxylase subunits converted aniline to 4-aminobenzoate with phenylphosphoamidate as intermediate under consumption of ATP. Only when both carboxylase subunits, one from gene cluster *ani* and the other from gene cluster *phe*, were combined, phenylphosphoamidate was converted to 4-aminobenzoate in vitro, with Mn^2+^, K^+^, and FMN as co-factors. Thus, aniline is degraded by the anaerobic bacterium *D. anilini* only by recruiting genes for the enzymatic machinery from different gene clusters. We conclude, that *D. anilini* carboxylates aniline to 4-aminobenzoate via phenylphosphoamidate as an energy rich intermediate analogous to the degradation of phenol to 4-hydroxybenzoate via phenylphosphate.

## Introduction

To date only few microorganisms are known that can degrade aniline. Several aerobic bacteria degrade aniline to the central intermediate catechol using oxygen-dependent reactions. Three enzymes are involved in the conversion of aniline to catechol: a glutamine synthetase-like enzyme produces glutamylanilide from glutamine and aniline, an aniline dioxygenase hydroxylates the aromatic ring and a glutamine amidotransferase-like enzyme prevents excessive accumulation of cytotoxic glutamylanilide by catalyzing the reverse reaction of the glutamine synthetase-like enzyme (Arora, 2015). The enzymes required in the aerobic degradation of aniline were characterized by heterologous expression of the enzymes in *E. coli* (Takeo et al., 2013). The key intermediate of aerobic aniline degradation, catechol, undergoes either *meta-*cleavage or *ortho-*cleavage, depending on the type of bacterium (Fujii et al., 1997; Fukumori and Saint, 1997; Murakami et al., 1998; Murakami et al., 2003; Liang et al., 2005; Xiao et al., 2009).

Under anoxic conditions, the aromatic ring cannot be activated by a dioxygenase because of the lack of oxygen. In the absence of oxygen, bacteria have to use completely different strategies to degrade aniline.

Aromatic degradation pathways in anaerobic bacteria are highly diverse and differ depending on the aromatic substrate and the available electron acceptor (Philipp and Schink, 2012). However, various anaerobic bacteria that degrade aromatic compounds generally share key intermediates of aromatic degradation (Fuchs et al., 2011). 4-aminobenzoate was identified as an intermediate in aniline metabolism of strain HY99, a close relative to *Delftia acidovorans*, during nitrate reduction (Kahng et al., 2000). In another anaerobic aniline-utilizing bacterium, *Desulfatiglans anilini*, aniline is oxidized with sulfate as electron acceptor (Schnell et al., 1989). It was postulated, that also in this sulfate-reducing bacterium 4-aminobenzoate is a key intermediate during aniline degradation (Schnell and Schink, 1991). Here, aniline was proposed to be carboxylated to 4-aminobenzoate, followed by activation of 4-aminobenzoate to 4-aminobenzoyl-CoA which is reductively deaminated to benzoyl-CoA (Schnell and Schink, 1991). Activities of 4-aminobenzoyl-CoA synthetase and benzoyl-CoA synthetase were observed in cell-free extracts of *D. anilini*, but the enzymes that convert aniline to 4-aminobenzoate have neither been identified nor functionally characterized. The objective of our study was to identify these enzymes.

Because phenol is structurally related to aniline it is conceivable that degradation of both compounds might be similar. The initial steps of phenol degradation comprise phenol activation to phenylphosphate by phenylphosphate synthase and subsequent carboxylation of phenylphosphate by phenylphosphate carboxylase in nitrate-, iron- or sulfate-reducing bacteria, regardless of the terminal electron acceptor (Schmeling et al., 2004; Schühle and Fuchs, 2004; Schleinitz et al., 2009; Xie and Müller, 2018). Although nitrate-, iron- or sulfate-reducing bacteria follow the same strategy to degrade phenol, different catalytic mechanisms of phenylphosphate carboxylase were adopted by these energy-limited bacteria. The nitrate-reducing bacterium *Thauera aromatica* harbors a phenylphosphate synthase which phosphorylates phenol with ATP and forms phenylphosphate and AMP (Schmeling et al., 2004). Phenylphosphate is then carboxylated with CO_2_ to 4-hydroxybenzoate by a phenylphosphate carboxylase that consists of four subunits (Schühle and Fuchs, 2004). The δ-subunit converts phenylphosphate to a phenolate intermediate, which in turn is carboxylated with CO_2_ to 4-hydroxybenzoate by the subunits αβγ (Schühle and Fuchs).

Subunit γ was described earlier to be unique to the phenylphosphate carboxylase in *Thauera aromatica*. The γ-subunit could not be overexpressed separately and required co-expression with the β-subunit in recombinant *E. coli*. Therefore, dispensability of the γ-subunit alone could not be tested. Both β- and γ-subunit showed slight decarboxylase activity when tested in reverse and for full activity, all four subunits are required in *T. aromatica* (Schühle and Fuchs, 2004).

The iron-reducing *Geobacter metallireducens* strain GS-15 and the sulfate-reducing *D. anilini* lack the gene coding for phenylphosphate carboxylase subunit γ (Schleinitz et al., 2009; Xie and Müller, 2018).

However, both *Geobacter metallireducens* strain GS-15 and *D. anilini* degrade phenol to 4-hydroxybenzoate with phenylphosphate as intermediate. The subunit composition of phenylphosphate synthase and phenylphosphate carboxylase appears to be different compared to *T. aromatica* (Schleinitz et al., 2009; Xie and Müller, 2018). Moreover, it was demonstrated that the phenylphosphate carboxylase subunits αβδ in *D. anilini* are recruited from different genomic loci during growth with phenol (Xie and Müller, 2018). We postulate that aniline is degraded analogously to phenol: Aniline is converted to phenylphosphoamidate which in turn is carboxylated to yield 4-aminobenzoate. Here, we present how *D. anilini* converts aniline to benzoyl-coenzyme A using differential proteome analysis and functional characterization of the key enzymes of the initial degradation steps.

## Materials and Methods

### Bacterial Growth Conditions

*Desulfatiglans anilini* was grown in anoxic bicarbonate-buffered (30 mM) and sulfide-reduced (2 mM) brackish water medium as described before (Xie and Müller, 2018). 1 mM of aniline, 4-aminobenzoate, benzoate or phenol, respectively, was added to the medium as sole source of carbon and energy. Sodium sulfate (5 mM) served as electron acceptor. The cultures were incubated at 30°C in anoxic cultivation bottles with butyl-rubber septa. For cloning *E. coli* NovaBlue plasmid transformed cells were grown aerobically in LB medium (10 g l^–1^ peptone, 5 g l^–1^ yeast extract, 10 g l^–1^ NaCl) at 37°C and 200 rpm/min with antibiotics (see plasmid construction and overexpression). For overexpression, *E. coli* Rosetta^TM^ 2 (DE3) was grown in anoxic medium, which was prepared from freshwater medium (Müller et al., 2009). Instead of adding sodium sulfide, 2 mM cysteine was added as reducing agent. In addition, the anoxic medium was supplemented with yeast extract (0.1 w/v), glucose (0.4% w/v), NaNO_3_ (50 mM), and the respective antibiotics.

### Preparation of Cell-Free Extracts of *D. anilini*

Cells of *D. anilini* were harvested in early stationary phase after an incubation time of one to 4 weeks depending of the substrate. The cultures reached an OD_600_ of 0.1 to 0.15 and were harvested by centrifugation at 8000 rpm for 30 min. Pellets were washed with 50 mM Tris–HCl buffer twice, followed by passing the cells through a French pressure cell (SLM Aminco, Cat. No. FA003, Urbana, IL, United States) three times at a pressure of 137 MPa to disrupt the cells. Then, cell debris was removed by centrifugation (30 min, 30,300 g, 4°C, Optima^TM^ TL Ultracentrifuge, Beckman Coulter, Brea, CA, United States). The supernatant was used for total proteome analysis.

### Total Proteome Analysis and Database Search

The cell-free extracts containing soluble proteins of *D. anilini* grown with different substrates (aniline, 4-aminobenzoate, benzoate or phenol, respectively), were submitted to the Proteomics Core Facility of the University of Konstanz for total proteome analysis. The protein digests were analyzed by using a LTQ Orbitrap Discovery with an Eksigent 2D-nano HPLC (Thermo Fisher Scientific, Waltham, MA, United States) or a Q-Exactive HF mass spectrometer (Thermo Fisher Scientific, Bremen, Germany) interfaced with an Easy-nLC 1200 nanoflow liquid chromatography system (Thermo Scientific, Odense, Denmark). The peptide digests were reconstituted in 0.1% formic acid and loaded onto the analytical column (75 μm × 15 cm). Peptides were resolved at a flow rate of 300 nL/min using a linear gradient of 6-40% solvent B (0.1% formic acid in 80% acetonitrile) over 75 min. Data-dependent acquisition with full scans in 350-1500 m/z range was carried out using the Orbitrap mass analyzer at a mass resolution of 120000 at 200 m/z. The 20 most intense precursor ions were selected for further fragmentation. Only peptides with charge states 2-6 were used, and dynamic exclusion was set to 30 s. Precursor ions were fragmented using higher-energy collision dissociation (HCD) with a normalized collision energy (NCE) set to 28%. Fragment ion spectra were recorded at a resolution of 15000. The Mascot search engine [Matrix Science, London, United Kingdom] was used to match each peptide fingerprint against the protein database of the IMG annotated genome of *D. anilini*. Relative protein abundances were expressed by the peak area of the peptides of each protein measured by the Eksigent 2D-nano total ion count (TIC). The term “area” here refers to the mean value of the two or maximally three most intense peptide intensities per protein, if at least two peptides per protein were identified. If no peptide was identified, the area value of the corresponding protein was set to zero. Due to the complexity of the protein mixtures, it might occur that some proteins were identified with two peptides in one sample but not in the replicate samples. The total proteomes of cell lysates of three independent cultures per cultivation condition (aniline, phenol, 4-aminobenzoate or benzoate) were analyzed. For the identification of differentially overabundant proteins, *Z*-scores were calculated for each individual protein modified after (Coombs et al., 2010) and using the standardization function in Microsoft Excel according to the following formula:

$$\text{Z-score}=\frac{\mathrm{area}\mathrm{of}\mathrm{protein}-\mathrm{average}\mathrm{of}\mathrm{all}\mathrm{areas}\mathrm{within}\mathrm{one}\mathrm{sample}}{\mathrm{Standard}\mathrm{deviation}\mathrm{of}\mathrm{all}\mathrm{areas}\mathrm{within}\mathrm{one}\mathrm{sample}}$$

In addition, the area values of aniline-grown cells versus phenol-grown cells were analyzed for significantly overabundant proteins as follows: Two-sided, paired *t*-tests were performed for each dataset per protein (three area values for aniline-grown cells and three area values for phenol-grown cells, respectively), with the *t*-test-function in Microsoft Excel which returns the two-sided *p*-value. The negative logarithms to base 10 of these *p*-values were plotted against the logarithm to base 2 of the ratio of the mean area value of aniline to phenol-grown cells (Volcano-plot, Paulo et al., 2013). Weakly significant proteins had a negative logarithm to base 10 of the *p*-value higher than 1.0 (*p* < 0.1), strongly significant proteins were higher than 1.3 (*p* < 0.05). The volcano plot was prepared as described before, but without the Benjamini-Hochberg correction for identifying false positives as described in the “Results” section (Paulo et al., 2013).

### Alignments of Amino Acid Sequences

Alignments of the amino acid sequences of the investigated genes were done using the Geneious software package (Version 11.1.5, Biomatters Ltd., Auckland, New Zealand). Alignments were calculated with the Multiple Alignment tool and the ClustalW program version 2.1 with the BLOSUM cost matrix, gap open cost adjustment 10 and gap extend cost adjustment 0.1 (Larkin et al., 2007).

### Phylogenetic Distance Tree

The phylogenetic tree was constructed using the MEGA software (Hall, 2013). First, the predicted amino acid sequences of the aniline-induced genes of *Desulfatiglans anilini* and closely related genes from other bacteria were downloaded from the JGI-IMG database^1^. Then, the above amino acid sequences were aligned using the ClustalW algorithm in MEGA. Finally, a phylogenetic tree from the aligned sequences was constructed by performing the neighbor-joining algorithm in MEGA.

### Plasmid Construction of Genes 03868, 03871, 03072 and 02059

All primers used for cloning are listed in Table 1. Genomic DNA of *D. anilini* was isolated from 10 ml of a dense culture (OD_600_ = 0.16) using the Gentra Puregene Cell Kit (Qiagen) following the manufacturer’s protocol. The genes 03871, 03868, 03872 and 02059 were amplified from genomic DNA of *D. anilini* by PCR using the primer-pairs 3871-F and 3871-R, 3868-F and 3868-R, 3872-F and 3872-R, 2059-F and 2059-R, respectively. The PCR mixture had a volume of 50 μl and contained 10 μl Phusion High Fidelity Polymerase buffer (New England Biolabs GmbH, Frankfurt am Main, Germany), 5 nmol dNTPs, 50 pmol of each primer, 10 to 50 ng of genomic DNA, and 0.5 μl Phusion High Fidelity Polymerase (2 U/μl). PCR amplification was performed using a T100 Thermal Cycler (Bio-Rad, Hercules, CL, United States). The PCR program consisted of an initial denaturation step at 94°C for 3 min, followed by 31 cycles of 94°C for 30 s, 60°C for 30 s, and 72°C for 1 min, and a final elongation step of 72°C for 5 min. The quality of PCR products was analyzed by electrophoresis in a 1.0% agarose gel at 110 V for 30 min and staining with ethidium bromide with a concentration of 0.5 μg/ml for 30 min. The gel was exposed to UV light and the picture of the gel was taken with a gel documentation system (Gel Doc^TM^ XR+ Gel Documentation System, Bio-Rad, CL, United States). Primers were ordered from Microsynth (Balgach, Switzerland). The DNA fragment generated by PCR using primers 3871-F and 3871-R was digested with *Sac I* and *Xho I*, and inserted into the same sites of restriction digested pET 28a, resulting in pXX 5 (Table 2). The DNA fragment generated by PCR using primers 3868-F and 3868-R was digested with *Sal I* and *Nde I*, and inserted into the same sites of pET 28a, resulted in pXX 6 (Table 2). The plasmid pcrscript/Mss I was linearized with *Mss I* and blunt-ligated with the PCR product of 03872 or 02059, resulting in pXX 7 or pXX 8, respectively. Plasmids pXX 7 or pXX 8 were digested with *Nde I* and *Sal I* yielding an approximately 1500 bp fragment, which was gel-purified with the Zymoclean Gel DNA Recovery kit (Zymo Research Europe, Freiburg, Germany) and inserted into the same sites of pET 28a, resulting in pFL 1 or pSS 1, respectively (Table 2). T4 DNA ligase (5 U/μl, Thermo Fisher Scientific, Waltham, MA, United States) was used for the ligation reactions following the manufacturer’s protocol. Two *Sal I* restriction sites were overseen in gene 02059, but the whole 1407 bp-gene was retrieved from incompletely digested pXX 8 by gel-purification, cloned into pET28a, and the sequence of the construct pSS 1 was confirmed to be correct. All ligation products were transformed into *E. coli* Nova Blue chemically competent cells. Positive clones were identified by colony PCR (Hanahan, 1983). The plasmids from positive colonies were purified using the QIAprep Spin mini kit (QIAGEN, Venlo, Netherlands) and submitted for sequencing to Microsynth (Balgach, Switzerland) to confirm the correct plasmid.

**TABLE 1:** Primers used for cloning in this study.

Primers’ name^a^	Primers’ sequence (5′-3′)^b^	Restriction enzymes
3871-F	CGGAGCTCATGCATTACGGAAGAA	*Sac I*
3871-R	CGGCTCGAGTTAGCTAATTTTATATACACGC	*Xho I*
3868-F	CCCATATGATGCTTGAGACAAGACCA	*Nde I*
3868-R	GCCGTCGACCTATTCATTAACTGGAAA	*Sal I*
3872-F	GCCCATATGATGAATGATCTTCGTTCA	*Nde I*
3872-R	GGCCGTCGACTTAAAATTTTGGTTCA	*Sal I*
2059-F	GCCCATATGATGAAAAGCATGAGAGATT	*Nde I*
2059-R	GCGTCGACTTAGAAACCCAGTTCAGA	*Sal I*

*^a^F or for, forward primer; R or rev, reverse primer. ^b^Restriction enzyme sites added in the primers were underlined and listed.*

**TABLE 2:** Plasmids used in this study.

Plasmids	Derivation and relevant characteristics^c^	Reference or source
pET 28a	Km^r^, pET 28a, carrying an *N*-terminal His Tag/thrombin/T7 Tag configuration plus an optional C-terminal His Tag sequence, can be used for expression of recombinant proteins in *E. coli*.	Merck Millipore
pcrscript/MssI	Ap^r^, pcrscript/MssI is a cloning vector carrying an ampicillin-resistance gene and multiple cloning site (MCS), which is modified from pPCR-Script Amp plasmid to include *Mss I* site in MCS.	Provided by Prof. Dr. Peter Kroth
pXX 5	Km^r^, pET 28a derivative containing gene 03871	This study
pXX 6	Km^r^, pET 28a derivative containing gene 03868	This study
pXX 7	Ap^r^, pcrscript/MssI derivative containing gene 03872	This study
pXX 8	Ap^r^, pcrscript/MssI derivative containing gene 02059	This study
pFL 1	Km^r^, pET 28a derivative containing gene 03872	This study
pSS 1	Km^r^, pET 28a derivative containing gene 02059	This study

*^c^Ap^r^, ampicillin resistance; Km^r^, kanamycin resistance.*

### Overexpression of Proteins Encoded by Genes 03868, 03871, 03872 and 02059

*E. coli* Rosetta 2 (DE3) cells (chloramphenicol resistance) were used to overexpress the recombinant proteins. Purified DNA plasmids (pXX 5, pXX 6, pSS 1 and pFL 1) were transformed chemically into *E. coli* Rosetta 2 (DE3) cells. Cells were grown in LB medium containing 50 μg/mL kanamycin and 35 μg/mL chloramphenicol over night at 37°C at 200 rpm. For anerobic overexpression, overnight cultures of *E. coli* containing the respective plasmid were inoculated into fresh LB medium containing 50 μg/mL kanamycin and 35 μg/mL chloramphenicol. When the OD_600_ reached 0.4 – 0.5, isopropyl β-D-1-thiogalactopyranoside (IPTG) was added to the culture at a concentration of 0.5 mM and the culture was further incubated at 37°C under shaking conditions (200 rpm) to induce protein expression. Samples were taken at time intervals (0, 2, and 4 h) to monitor the overexpression of proteins.

For anoxic overexpression, an overnight culture of *E. coli* containing the respective plasmid was inoculated into anoxic freshwater medium with 50 μg/mL kanamycin and 35 μg/mL chloramphenicol and grown at 37°C in a vertical shaker at 200 rpm until an OD_600_ of 0.4 – 0.5 was reached. Then, IPTG (0.2 mM) was added to the culture. After induction the anoxic cultures were incubated at 15°C overnight without shaking for protein expression. Samples were taken anaerobically to monitor the overproduction of proteins.

### Refolding of the Proteins

Purification of the heterologously overproduced proteins was performed under strictly anoxic conditions in a glove box (Coy, Ann Arbor, MI, United States). *E. coli* cells were harvested by centrifugation at 7000 rpm for 30 min in a centrifuge (Dupont Sorvall, Midland, Canada). Pellets were washed with 50 mM Tris–HCl buffer (pH 7.5) twice and finally resuspended in 5 mL 50 mM Tris–HCl buffer (pH 7.5), followed by passing the cells through a French pressure cell (SLM Aminco, Cat. No. FA003, Urbana, II, United States) three times at a pressure of 137 MPa applied by a French pressure cell press (SLM Aminco, Urbana, II, United States) for cell-disruption in anoxic serum vials sealed with butyl-rubber stoppers.

The preparation of inclusion bodies and refolding of proteins followed the procedures described by Schmeling et al. (2004). Inclusion bodies were obtained from the cell-free extracts by centrifugation at 5000 *g* for 10 min at 4°C. Then the pellets containing inclusion bodies were washed sequentially with two washing buffers, washing buffer 1 (2 ml, 50 mM Tris-Cl, pH 8.0, 1 mM EDTA, 1% (vol/vol) Triton X-100) and washing buffer 2 (2 ml, 50 mM Tris-Cl, pH 8.0, 1 mM EDTA, 0.5 M urea). The inclusion bodies were solubilized in 0.5 ml urea solution (100 mM Tris-Cl, pH 8.5, 8 M urea, 50 mM mercaptoethanol) by stirring for 1 h on ice. The soluble proteins were present in the supernatant after centrifugation at 40,000 *g* for 30 min. The supernatant was added dropwise into 4.5 ml ice-cold refolding solution (15% (vol/vol) glycerol, 50 mM Tris-Cl, pH 8.5, 10 mM mercaptoethanol) while gently stirring on ice. All operations were performed under strictly anoxic conditions in a glove box (Coy, Ann Arbor, MI, United States). After these treatments, the final concentration of the refolded proteins was approximately 3 mg/ml and the urea concentration was reduced to 0.8 M. The protein concentration was measured with the Bradford assay using bovine serum albumin as protein standard (Bradford, 1976). The purity of the refolded proteins was analyzed by one-dimensional denaturing polyacrylamide gel electrophoresis (SDS-PAGE).

### Identification of Protein Expression

Samples of each 1 ml of culture from *E. coli* for protein overexpression taken during induction with IPTG were treated with two methods. To obtain total proteins, samples were centrifuged at 14,000 rpm for 10 min and pellets were resuspended in 60 μl polyacrylamide gel electrophoresis (SDS-PAGE) loading buffer and cells opened by heating at 99°C for 10 min. Total protein was used for SDS-PAGE analysis. To obtain soluble proteins, 1 ml samples of *E. coli* cultures were harvested by centrifugation at 14,000 rpm for 10 min, resuspended in 30 μl 50 mM Tris–HCl buffer with 5 μg/ml lysozyme and incubated at 37°C for 1 h. Then, non-lysed cells and cell debris was removed by centrifugation (14,000 rpm, 10 min), and 30 μl SDS-PAGE loading buffer was added to the supernatant and used for SDS-PAGE analysis.

SDS-PAGE was performed to analyze overexpression, solubilization and purification of proteins. The denaturing polyacrylamide gel consisted of a 4% stacking gel and a 12% resolving gel (Laemmli, 1970). Gels were run at a constant current of 20 mV per gel for 1.5 h in running buffer (24.8 mM Tris, 192 mM glycine and 3.47 mM SDS). Proteins in gels were stained by incubation in colloidal Coomassie staining solution containing 2% H_3_PO_4_, 6% (NH_4_)_2_SO_4_, 20% methanol, and 0.08% (w/v) Coomassie Brilliant Blue R-250 overnight (Neuhoff et al., 1988) and washed in distilled water. Protein concentrations were estimated with the Bradford assay using bovine serum albumin as protein standard (Bradford, 1976).

### Enzyme Assays

The refolded proteins were used in enzyme assays and all enzyme assays were performed in cuvettes sealed with rubber stoppers and previously flushed with 100% nitrogen at 30°C under strictly anoxic conditions.

Phenylphosphoamidate synthase and phenylphosphoamidate carboxylase enzyme assays: the standard enzyme assay mixture contained 50 mM KPP buffer (pH 7.5, 0.5 mM DTT), 2 mM MnCl_2_, 2 mM KCl, 30 mM NaHCO_3_, 0.2 mM flavin mononucleotide (FMN), 2 mM ATP, 2 mM MgCl_2_, 2 mM aniline and 0.5 mg of each protein (Pasβ, Pasα, Ppcβ and Ppcβ2). To test the feasibility of ADP, 2 mM ADP was added to the standard assay system to replace ATP. To test the activity with CO as possible aniline carbonylation agent, 10% CO was added to the standard assay system to replace 30 mM NaHCO_3_. For control assays, no NaHCO_3_ was added to the standard assay system. To test the enzyme activity with phenol as substrate, 2 mM phenol was added to the standard assay system to replace aniline.

Phenylphosphoamidate synthase enzyme assays: the standard enzyme assay mixture contained 50 mM KPP buffer (pH 7.5, 0.5 mM DTT), 2 mM ATP, 2 mM MnCl_2_, 2 mM KCl, 2 mM MgCl_2_, 1 mM aniline and 0.5 mg of each protein (03871 and 03868).

Phenylphosphoamidate carboxylase enzyme assays: The standard enzyme assay mixture contained 50 mM KPP buffer (pH 7.5, 0.5 mM DTT), 2 mM MnCl_2_, 2 mM KCl, 30 mM NaHCO_3_, 0.2 mM flavin mononucleotide (FMN), 2 mM phenylphosphoamidate and 0.5 mg of each protein (03872 and 02059). To test the feasibility of CO, 10% CO was added to the standard assay system to replace 30 mM NaHCO_3_.

To analyze the reaction product, 200 μl samples were withdrawn at time intervals and the reaction was stopped by addition of an equal volume of acetonitrile and centrifuged (11,700 *g* for 10 min). The supernatant was transferred to 200 μl HPLC vials and analyzed by liquid chromatography-mass spectrometry.

### Liquid Chromatography-Mass Spectrometry

Samples of the enzyme assays were analyzed using a Waters Acquity UPLC system connected to a ThermoFisher Exactive Orbitrap high resolution mass spectrometer fitted with a heated electrospray ion source (HESI). The mass spectrometer was operated in positive ionization mode at 50000 resolution with internal calibration to the lock mass 455.12002. For UPLC separation a Macherey Nagel Nucleodur Sphinx RP column (100 × 2 mm, 1.8 μm) was used. HPLC program: 2% B 0 min, in 5 min to 100% B, 3 min 100% B, in 0.5 min to 2% B, 2.5 min 2% B, solvent A: H_2_O 0.1% AcOH, solvent B: MeOH 0.1% AcOH, flow rate: 0.3 ml/min. 0.2–1 ul of the samples was injected. Aniline: 1.5 min, [M+H]^+^ m/z 94.06525, Δppm 1.3, C_6_H_8_N; phenylphosphoamidate: 4.0 min, [M+H]^+^ m/z 174.03140, Δppm 0.32, C_6_H_9_O_3_NP; 4-aminobenzoate: 2.9 min, [M+H]^+^ m/z 138.05498, Δppm 0.18, C_7_H_8_O_2_N, phenylphosphate: 4.5 min, [M+H]^+^ m/z 175.01553, C_6_H_8_O_4_P, Δppm 0.33.

### Synthesis of Phenylphosphoamidate

Phenylphosphoamidate was synthesized via the intermediates tribenzyl phosphite, dibenzyl *N*-phenylphosphoramidate and triethylammonium *N*-phenylphosphoramidate.

Tribenzyl phosphite (Gefflaut et al., 1997) (1): A solution of phosphorus trichloride (7.87 g, 57.3 mmol) in diethylether (300 mL) was prepared under inert nitrogen atmosphere. To the stirred solution was added dropwise a solution of triethylamine (18.20 g, 180 mmol) in diethylether (30 mL), followed by benzyl alcohol (18.7 g, 173.1 mmol) in diethylether (30 mL). The reaction mixture was stirred 30 min at −40°C and 48 h at room temperature. The slurry of tribenzyl phosphite triethylammonium chloride was filtered through a sintered glass funnel under strict exclusion of oxygen. After solvent removal, tribenzyl phosphite (1) was isolated as slightly yellow oil in 86% yield (17.3 g) which was sufficiently pure for the next reaction step.^ 1^H-NMR (400 MHz, DMSO): δ 4.88 (d, *^3^J* = 8.1 Hz, 6H, CH_2_), 7.25–7.40 (m, 15H, H_ar_); ^31^P-NMR (162 MHz, DMSO): δ 135.5.

Dibenzyl *N*-phenylphosphoramidate (Michalski et al., 1980) (2): A solution of tribenzyl phosphite (1) (17.27 g, 49.0 mmol) in dichloromethane (80 mL) was prepared under nitrogen atmosphere. To the stirred and cooled (0°C) solution was added dropwise bromine (7.48 g, 46.8 mmol) in dichloromethane (60 mL). The mixture was stirred for 1 h at 0°C, then 25 min at room temperature and finally transferred dropwise *via canula* to a precooled (−40°C) mixture of aniline (6.63 g, 71.2 mmol) dissolved in 120 mL dichloromethane. Stirring was continued for 2 h at −40°C, then for an additional 12 h at room temperature. After solvent removal the residue was taken up in diethylether (500 mL). The solution was consecutively washed with 2 M HCl (2 × 150 mL, 1 × 100 mL), sodium thiosulfate solution (1 × 150 mL) and finally with brine (1 × 150 mL). The organic layer was dried over MgSO_4_, evaporated to dryness and purified by chromatography on silica gel with a petrol ether/ethyl acetate gradient-system (PE:EE 3:1 →1:1). Dibenzyl *N*-phenylphosphoramidate (synthesis reaction 2) was isolated as off-white solid in 78% yield (13.51 g). ^1^H-NMR (400 MHz, CDCl_3_): δ 5.06, 5.16 (AB part of ABX, ^2^*J*_HH_ = 11.7 Hz, ^3^*J*_HP_ = 7.5 Hz, 4H, OCH_2_-Ph), 6.93–6.99 (m, 1H_para_, N-C_6_H_5_), 7.00–7.05 (m, 2H_ortho_, N-C_6_H_5_), 7.17–7.25 (m, 2H_meta_, N-C_6_H_5_), 7.30 (s, 10H, OCH_2_C_6_H_5_); ^13^C-NMR (101 MHz, CDCl_3_): δ 68.35 (d, *J* = 4.6 Hz, CH_2_), 117.66 (d, *J* = 7.4 Hz, C2-aniline), 121.77 (C4-aniline), 128.01 (C2-Ph), 128.38 (C4-Ph), 128.47 (C3-Ph), 129.23 (C3-aniline), 135.72 (d, *J* = 7.9 Hz, C1-Ph), 139.43 (C1-aniline); ^31^P-NMR (162 MHz, CDCl_3_):δ – 0.17.

Triethylammonium *N*-phenylphosphoramidate (Chanley and Feageson, 1958; Burlingham and Widlanski, 2001) (3): Dibenzyl *N*-phenylphosphoramidate (synthesis reaction 2) (510 mg, 1.44 mmol) and Pd catalyst (190 mg, 10% Pd/C) were suspended in ethanol (10 mL) containing triethylamine (0.5 g). The flask was evacuated and backfilled with hydrogen 4 times. After 1 h hydrogenation the flask was evacuated and backfilled with nitrogen, the catalyst was removed by filtration over a bed of celite and the solvent removed under vacuum. Triethylammonium *N*-phenylphosphoramidate was isolated in quantitative yield (393 mg) and sufficiently pure for the *in vitro* enzyme assays.^ 1^H-NMR (400 MHz, CD_3_OD): δ 1.29 (t, *J* = 7.2 Hz, 9H, CH_3_), 3.12 (q, *J* = 7.2 Hz, 6H, CH_2_), 6.71 – 6.79 (m, 1H, aniline), 7.04 – 7.19 (m, 4H, aniline); ^13^C NMR (101 MHz, CD_3_OD): δ 9.04 (CH_3_), 47.34 (CH_2_), 117.84 (d, *J* = 7.0 Hz, C2-aniline), 119.92 (C1-aniline), 129.65 (C3-aniline), 144.94 (C1-aniline); ^31^P-NMR (162 MHz, CD_3_OD): δ –3.73.

The NMR spectra of phenylphosphoamidate and its precursors are shown in the (Supplementary Figures S1–S8).

### Stability of Phenylphosphoamidate

1 mM phenylphosphoamidate was dissolved in distilled water (pH 7.0) or Tris–HCl buffer (pH 8.0). The solutions were kept at room temperature 25°C. The degradation of phenylphosphoamidate was followed over time using a reversed-phase HPLC system (Shimadzu, Kyoto, Japan), which was equipped with a diode array detector and a Phenomenex Synergi Max-RP column (250 × 4.6 mm, 80Å, 4 um) (Phenomenex, Torrance, CA, United States). Eluents were prepared by mixing ultrapure water with 0.1% H_3_PO_4_ (buffer A), and acetonitrile with 0.1% H_3_PO_4_ (buffer B) and filtration through 0.2 μm. Isocratic elution at 90% buffer A was used at a flow rate of 0.8 ml min^–1^ at 25°C. 25 μl were injected into the column. The formation of aniline was observed based on the retention time and its UV-absorption at 235 and 287 nm.

### Chemicals

All chemicals were of analytical quality and, except phenylphosphoamidate, were obtained from Merck/Sigma-Aldrich (Darmstadt, Germany). Gases were purchased from Messer-Griesheim (Darmstadt, Germany) and Sauerstoffwerke Friedrichshafen (Friedrichshafen, Germany).

## Results

### Differential Proteome Analysis of *D. anilini* Grown With Aromatic Compounds as Sole Carbon Source

**FIGURE 1:**
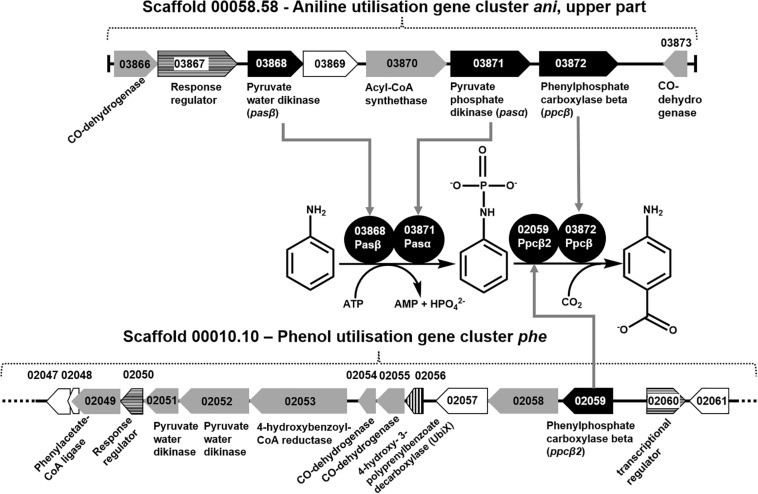
Organization of gene cluster *ani* (upper part) and gene cluster *phe*, as well as the recruitment of the proteins necessary for conversion of aniline to 4-aminobenzoate. Black gene arrows: genes heterologously expressed in *E. coli* and assayed in this study; gray gene arrows: genes of catalytic enzymes putatively involved in the degradation of aniline, 4-aminobenzoate, phenol, or benzoate; white gene arrows: genes for hypothetical proteins or uncharacterized proteins; white gene arrows with horizontal line fill: putative regulatory genes; gene arrow with vertical line fill: ubiX-like gene for 4-hydroxy-3-polyprenylbenzoate-decarboxylase.

**FIGURE 2:**
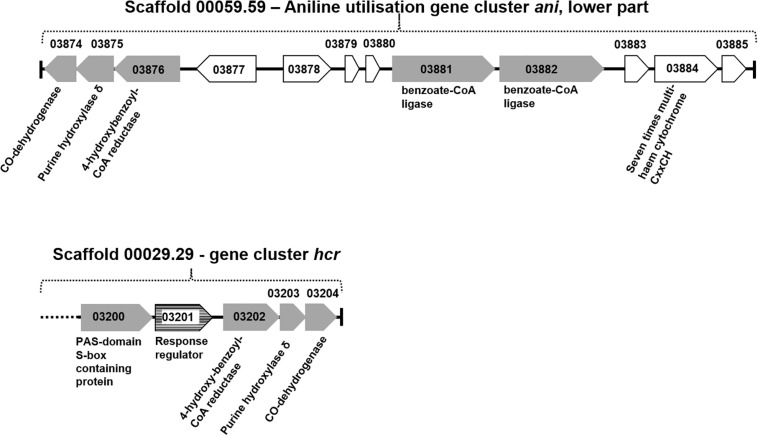
Organization of gene cluster *hcr* and gene cluster *ani* (lower part). Gray gene arrows: genes of catalytic enzymes putatively involved in the degradation of aniline, 4-aminobenzoate, phenol, or benzoate; white gene arrows: genes for hypothetical proteins or uncharacterized proteins; white gene arrows with horizontal line fill: putative regulatory genes.

**FIGURE 3:**
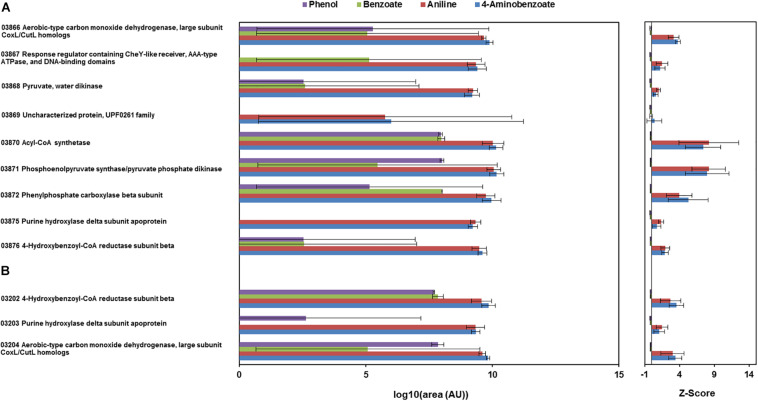
Total proteome analysis of cell-free extracts from the cells of *D. anilini* grown on phenol (purple bars), benzoate (green bars), aniline (red bars) and 4-aminobenzoate (blue bars), as sole carbon source, respectively. Protein abundances are represented as area values of the MS-signals of peptides identified with the Proteome Discoverer software. The logarithm to the base 10 of mean values and standard deviations of the protein abundances (area) in cell-lysates from three independent cultures under the respective growth condition are shown (AU: arbitrary units). *Z*-scores are depicted as a standardized representation of the data. Positive *Z*-scores represent values above the average, negative *Z*-scores represent values below the average. The highest observed *Z*-score was 27.1 (H567DRAFT_02821 dissimilatory adenylylsulfate reductase alpha subunit precursor) and the lowest observed *Z*-score was -0.23 (H567DRAFT_04108 Hemerythrin-like domain-containing protein). **(A)** Abundances of the enzymes encoded by gene cluster *ani* (locus tag 03866-03876). **(B)** Abundances of the enzymes encoded by gene cluster *hcr* (locus tag 03202-03204).

The total proteomes of *D. anilini* grown with four different sole carbon sources (phenol, benzoate, aniline, and 4-aminobenzoate), respectively, were compared semi-quantitatively. The proteins that were highly abundant after growth with aniline or 4-aminobenzoate were analyzed: Among them, the proteins encoded by a putative gene cluster identified in this study, which we termed gene cluster *ani*, owing to the fact, that the corresponding genes were higher expressed during growth with aniline or 4-aminobenzoate compared to growth with phenol or benzoate as judged by their *Z*-scores (locus tag from H567DRAFT_03866 to H567DRAFT_03876 (Figures 1–3). Four proteins (among them 4-hydroxy-benzoyl-CoA reductase) of genes with different genomic locations in a putative gene cluster also identified in this study, which was termed gene cluster *hcr*, were at least ca. 100-fold more abundant and had higher *Z*-scores above the average in aniline- and 4-aminobenzoate-grown cells compared to phenol- or benzoate-grown cells (Figures 2, 3B, locus tag from H567DRAFT_03202 to H567DRAFT_03204). Three proteins, namely H567DRAFT_03871, H567DRAFT_03872, and H567DRAFT_03868, were of particular interest because their annotations suggested their involvement in phosphorylation and carboxylation of aromatic compounds (Figure 1). The gene of the protein with the highest abundance in the four investigated growth conditions was annotated as dissimilatory adenylylsulfate reductase α-subunit precursor (H567DRAFT_02821, Supplementary Table S1). Phenylphosphate carboxylase β-subunit (H567DRAFT_02059) was also most abundant during growth with phenol as judged by its *Z*-score (Supplementary Figure S9, Supplementary Table S1). The latter protein has been described previously to be involved in phenol carboxylation in *D. anilini* (Xie and Müller, 2018). In the following, H567DRAFT_ will be omitted for the gene identification and only the last digits will be used. The gene for phenylphosphate carboxylase β-subunit (*ppcβ2* 02059) is located in another putative gene cluster, which we termed gene cluster *phe* in this study. Besides *ppcβ2*, the putative gene cluster *phe* contains other genes for phenol-degrading enzymes as described previously (Figure 1; Xie and Müller, 2018). The proteome data of triplicates from independent cultures was also analyzed for significantly overabundant proteins in aniline- versus phenol-grown cells in a volcano plot (Supplementary Figure S10). From all proteins tested by a *t*-test, 117 had a *p*-value lower than 0.1 and 59 proteins had a *p*-value lower than 0.05. These proteins were therefore considered significantly higher abundant under aniline-grown conditions. Of these 117 proteins, 11 had a log 2-fold overabundance of at least 2, and were considered likely candidates for proteins, that were specifically produced in aniline-grown cells. Three of these 11 proteins are annotated as aerobic-type carbon monoxide dehydrogenase subunits of which two are located in the putative gene clusters *ani* and *phe*, respectively (03204, 03866). Other highly abundant and at least weakly significant proteins are a competence protein, one type VI secretion system protein, one hemerythrin- like domain-containing protein, one pyruvate water dikinase (03868, gene cluster *ani*), one pyruvate phosphate dikinase (03871, gene cluster *ani*), one phenylacetate-CoA ligase and two TRAP-type C4-dicarboxylate transport system substrate-binding proteins (Supplementary Figure S10).

The proteomes of aniline-grown cells and 4-aminobenzoate-grown cells did not differ significantly from each other. For the *ani* gene cluster (locus tags from 03866 to 03876), the organization is shown in Figures 1, 2. The *ani* gene cluster comprises genes coding for a pyruvate water dikinase/phosphoenolpyruvate synthase (03868) and a phosphoenolpyruvate synthase/pyruvate phosphate dikinase 03871). The latter two are highly overabundant in aniline- versus phenol-grown cells, but with weak significance (*p* < 0.1, Supplementary Figure S10). BLAST-search of the respective amino acid sequences revealed that both genes exhibited high identities to phenylphosphate synthase subunits of the nitrate-reducing *Thauera aromatica* K172*:* phenylphosphate synthase β (Ppsβ, 41%) and phenylphosphate synthase subunit α (Ppsα, 28%). Thus, the proteins of genes 03868 and 03871 were suspected to activate aniline to phenylphosphoamidate and are therefore termed phenylphosphoamidate synthase Pasα (03871) and Pasβ (03868). Phenylphosphate carboxylase β-subunit (03872) was the candidate protein to carboxylate phenylphosphoamidate to 4-aminobenzoate. This protein was also highly overabundant in aniline- versus phenol-grown cells, but the *t*-tests showed a low significance (*p* = 0.116, Supplementary Figure S10, Figure 3). The annotations of the other genes in gene clusters *ani* and *phe* suggest, that they are involved in the downstream degradation pathway of aniline or phenol in *D. anilini*, which was not investigated in this study. Another set of genes further downstream in gene cluster *ani* is annotated as 4-hydroxybenzoyl-CoA reductase and purine hydroxylase delta subunit apoprotein (03875 and 03876, Figure 2). The proteins of these genes might play a role in reductive deamination of 4-aminobenzoyl-CoA but their function was not in the focus of this study. The respective proteins were abundant only during growth with 4-aminobenzoate or aniline and not observed at all during growth with benzoate or phenol (Figure 3, Supplementary Table S1). Proteins of another homologous set of genes that are annotated as 4-hydroxybenzoyl-CoA reductase and purine hydroxylase and located in another area of the genome, (gene cluster *hcr*, 03202 and 03203) were also observed only when *D. anilini* was grown with aniline (Figure 3B, Supplementary Table S1).

The lower part of gene cluster *ani* also contains genes for two benzoate-CoA ligases (03881 and 03882). These genes were constitutively present under all tested growth conditions, except for 03881 which was not expressed during growth with aniline (Supplementary Table S1).

The abundance of enzymes involved in phenol degradation in *D. anilini* was also monitored for the cells grown with aniline, 4-aminobenzoate, benzoate or phenol as sole carbon source. The phenol-degrading enzymes are encoded in a gene cluster (gene locus tag from 02049 to 02059). The phenol-degrading enzymes are highly induced in phenol-grown cells, but not in benzoate-grown cells (Supplementary Figure S9, Xie and Müller, 2018). Supplementary Figure S9 shows that phenol-degrading enzymes were also produced at a moderate level in aniline and 4-aminobenzoate-grown cells. The putative phenylphosphate carboxylase β-subunit (02059) was most abundant in phenol grown cells, but also somewhat abundant in aniline-grown cells (*Z*-score phenol: 8, *Z*-score aniline: 0.15, Supplementary Figure S9, Supplementary Table S1). The other two subunits of phenylphosphate carboxylase (α-subunit, 03563, δ-subunit, 00862) had *Z*-scores below the average during growth with aniline (α-subunit phenol: 4.25, α-subunit aniline: −0.04, δ-subunit phenol: −0.23, δ-subunit aniline: −0.22, Supplementary Figure S9, Supplementary Table S1). This indicated that *D. anilini* mainly uses different enzymes to degrade phenol and aniline (or 4-aminobenzoate). However, the abundant phenylphosphate carboxylase β-subunit (02059) of the phenol-degrading gene cluster appeared to be potentially involved both in phenol and aniline degradation, even though the log 2-fold aniline versus phenol abundance was negative (Supplementary Figure S10). Alignment of the amino acid sequences of the respective aniline- and phenol-degrading enzymes (Supplementary Figures S11–13) revealed, that the phenylphosphate synthase α-subunit (Ppsα) and phenylphosphoamidate synthase α subunit (Pasα) are only distantly related with an identity of 26.73%. The highly abundant phenylphosphate carboxylase β-subunit (Ppcβ2) encoded in gene cluster *phe* and the protein of the gene annotated as phenylphosphate carboxylase β-subunit (Ppcβ, 03872) in gene cluster *ani* are more closely related and have an amino acid identity of 51.71% (alignment not shown).

### Heterologous Overexpression and Refolding of Proteins

In order to establish the function of the candidate aniline-degrading enzymes identified by differential proteomics the genes from gene cluster *ani* coding for the proteins pyruvate water dikinase (*pasβ*), phosphoenolpyruvate synthase/pyruvate phosphate dikinase (*pasα*), and phenylphosphate carboxylase β-subunit (*ppcβ*) were cloned and overproduced in *E. coli.* The presence of only one carboxylase subunit in gene cluster *ani* suggested, that other subunits of phenylphosphate carboxylase might be important as well, as during growth with phenol, three phenylphosphate carboxylase subunits (αβδ) from different genomic loci are required by *D. anilini* (Xie and Müller, 2018). As the phenylphosphate carboxylase subunits α and δ were identified in the proteome at comparably low levels, we only investigated the potential role of the abundant *ppcβ2* in addition to the genes from gene cluster *ani*.

**FIGURE 4:**
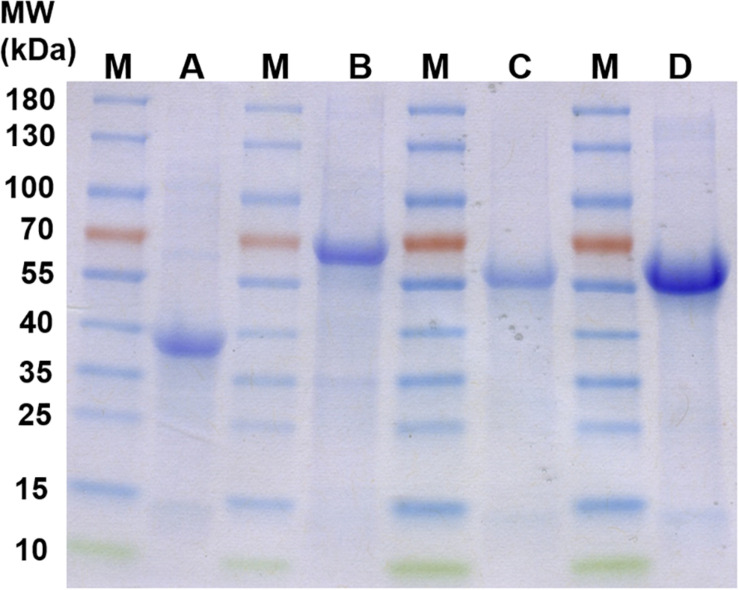
SDS-PAGE gels of recombinant, refolded proteins. M: Molecular mass-marker (Thermo Fisher Scientific, PageRuler^TM^ Prestained Protein Ladder). A: Refolded protein Pasβ (3868). B: Refolded protein Pasα (3871). C: Refolded protein Ppcβ (3872). D: Refolded protein Ppcβ2 (2059). Each of the lanes A to D contained 15 μg total protein.

The proteins Pasα (03871), Pasβ (03868), Ppcβ (03872), and Ppcβ2 (02059) were overproduced in *E. coli* Rosetta 2 DE3. The overproduced proteins were not soluble when the cells were grown in LB medium aerobically at 37°C and induced by addition of 0.5 mM isopropyl β-D-1-thiogalactopyranoside (IPTG) (data not shown). Alternatively, anoxic medium and addition of ethanol (Thomas and Baneyx, 1997) were tried to obtain soluble protein. Unfortunately, no soluble protein was obtained under any growth condition tried. All overproduced proteins were present in inclusion bodies. The insoluble proteins were therefore solubilized with Triton X-100, urea and refolded. Figure 4 shows the refolded proteins Pasβ, Pasα, Ppcβ and Ppcβ2 on a SDS-PAGE gel with a molecular weight of approximately 39, 63, 53, and 52 KDa, respectively.

### Functional Analysis of Phenylphosphoamidate Synthases (Pasα and Pasβ), and Phenylphosphoamidate Carboxylases Ppcβ and Ppcβ2

**FIGURE 5:**
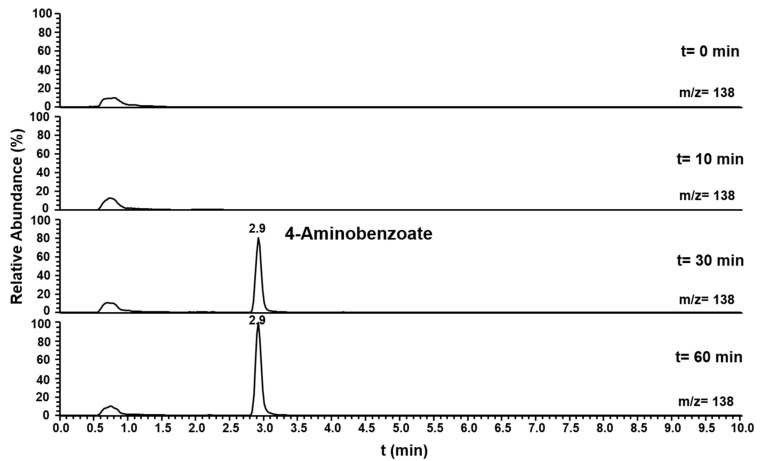
Ion trace chromatograms showing the time course of 4-aminobenzoate formation from aniline by the heterologuously expressed phenylphosphoamidate synthase and phenylphosphoamidate carboxylase from *D. anilini*. The assay contained the subunits Pasα, Pasβ, Ppcβ, and Ppcβ2.

Pasα and Pasβ were predicted to be a pyruvate water dikinase and a pyruvate phosphate dikinase, suggesting that these two proteins catalyze the phosphorylation of the amino group of aniline to form phenylphosphoamidate, in analogy to the phosphorylation of phenol to phenylphosphate by phenylphosphate synthase in *Thauera aromatica* K172 (Schmeling et al., 2004). Ppcβ and Ppcβ2 were predicted to be phenylphosphate carboxylase β-subunits, suggesting that these enzymes are involved in the carboxylation of phenylphosphoamidate to 4-aminobenzoate. A combined assay (aniline phosphorylation plus phenylphosphoamidate carboxylation) with Pasα, Pasβ, Ppcβ, and Ppcβ2 was performed with aniline and NaHCO_3_ as substrates and ATP, Mg^2+^, Mn^2+^, K^+^ and FMN as co-factors. The formation of 4-aminobenzoate from aniline as substrate (Figure 5) was observed by LC-MS. NaHCO_3_ could not be replaced by CO. The intermediate phenylphosphoamidate could not be observed by LC-MS most likely because phenylphosphoamidate is very unstable in aqueous solution and as an energy-rich intermediate accumulates only to low concentrations (Supplementary Figure S14). When ADP was employed as an alternative phosphate group donor to replace ATP, no formation of 4-aminobenzoate could be detected.

**FIGURE 6:**
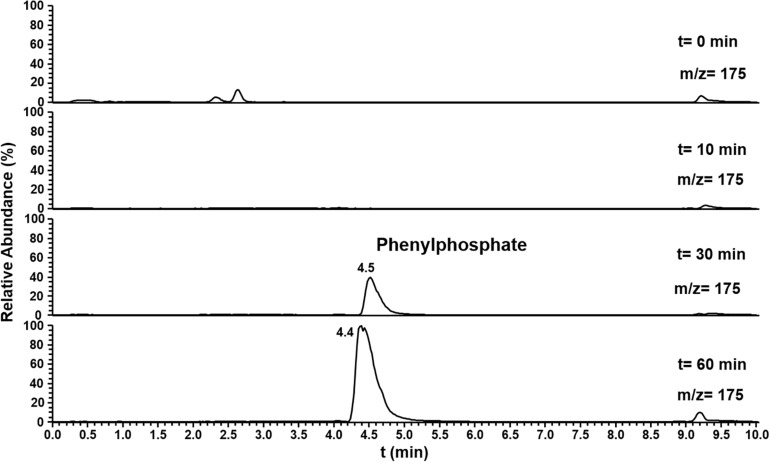
Ion trace chromatograms showing the time course of phenylphosphate formation from phenol by the heterologuously expressed phenylphosphoamidate synthase from *D. anilini*. The assay contained the subunits Pasα and Pasβ.

The substrate specificity of phenylphosphoamidate synthase and phenylphosphoamidate carboxylase was challenged offering phenol as substrate. Here the formation of phenylphosphate from phenol by phenylphosphoamidate synthase was observed by LC-MS after 30 min (Figure 6). However, phenylphosphoamidate carboxylase did not convert phenylphosphate to 4-hydroxybenzoate.

### Functional Analysis of Phenylphosphoamidate Synthase Pasα and Pasβ

The enzyme activity of phenylphosphoamidate synthase (Pasα and Pasβ) was tested by following the conversion of aniline to phenylphosphoamidate in the presence of aniline, Mg^2+^, K^+^, Mn^2+^ by Pasα and Pasβ. However, it was not possible to observe the formation of phenylphosphoamidate directly because phenylphosphoamidate decomposes too quickly (Supplementary Figure S14). The subunits Pasα and Pasβ were only employed in enzyme assays together and the activities of the single subunits alone was not tested in separate assays.

### Functional Analysis of Phenylphosphoamidate Carboxylase (Ppcβ and Ppcβ2)

**FIGURE 7:**
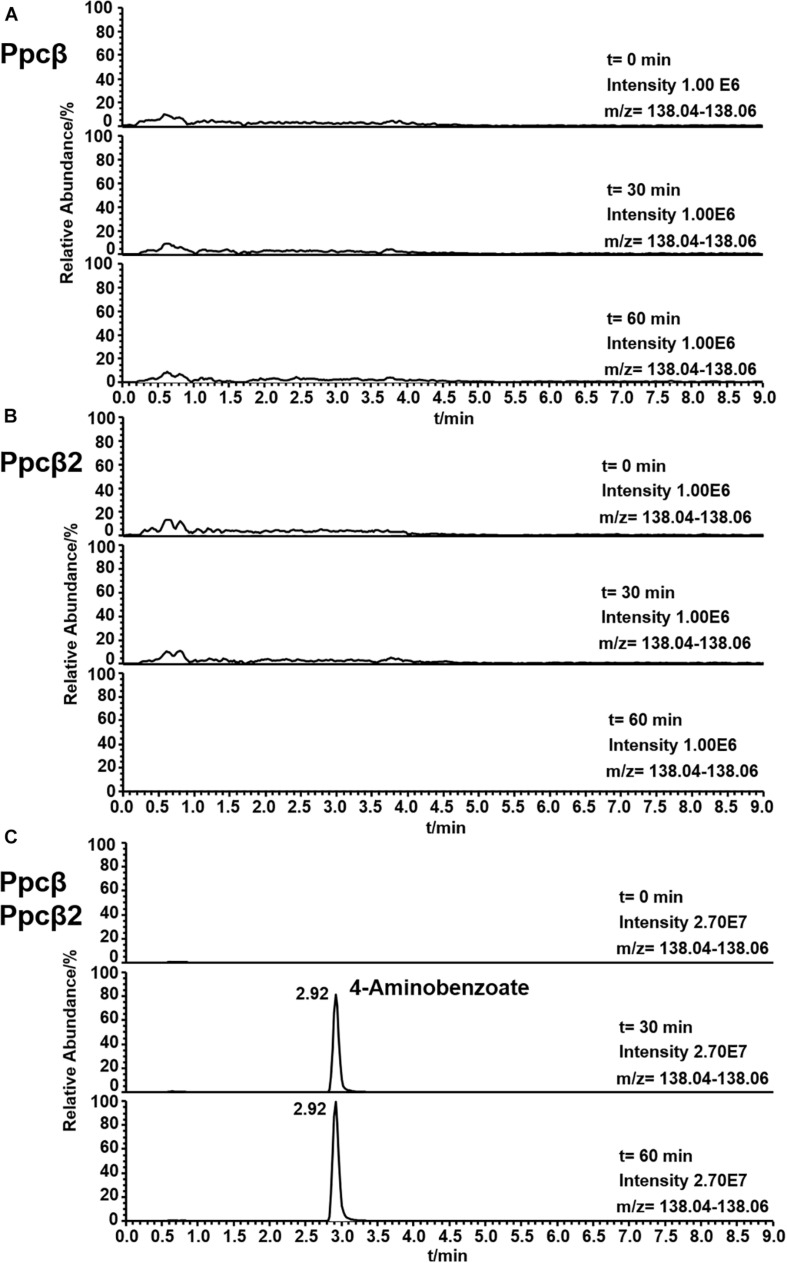
Ion trace chromatograms showing the time course of 4-aminobenzoate formation from phenylphosphoamidate by the heterologuously expressed phenylphosphoamidate carboxylase from *D. anilini*. The time courses of enzyme assays of each individual subunit Ppcβ alone **(A)**, subunit Ppcβ2 alone **(B)**, as well as of Ppcβ and Ppcβ2 **(C)** are presented.

The enzyme activity of phenylphosphoamidate carboxylase (Ppcβ and Ppcβ2) was followed by observing the formation of 4-aminobenzoate from phenylphosphoamidate. Figure 7 shows the formation of 4-aminobenzoate from phenylphosphoamidate in an assay consisting of proteins of genes 03872 and 02059 (Ppcβ and Ppcβ2), phenylphosphoamidate, NaHCO_3_ and the co-factors FMN, Mg^2+^, Mn^2+^, and K^+^. No activity could be measured in the absence of the co-substrate NaHCO_3_. Carbon monoxide (CO) was also tested as a potential co-substrate in the enzyme assay, but no enzyme activity was detected. The enzyme needs Mn^2+^ and K^+^ ions for activity. Ppcβ and Ppcβ2 were tested individually in order to evaluate if one enzyme is sufficient to convert phenylphosphoamidate to 4-aminobenzoate. The single enzymes did not catalyze the formation of 4-aminobenzoate from phenylphosphoamidate. Thus both enzymes are essential for the conversion of phenylphosphoamidate to 4-aminobenzoate (Figure 7).

## Discussion

Differential proteomics indicated that at least three gene clusters are involved in aniline degradation (*ani* gene cluster and *hcr* gene cluster, as well as the gene coding for an aromatic carboxylase subunit (*ppcβ2*, 02059) of the phenol degradation *phe* gene cluster (Figures 1, 2). Interestingly, *D. anilini* uses genes from different locations in the genome to constitute the enzymes needed for the degradation of aniline. Because the enzymes involved in the initial steps of phenol degradation and aniline degradation perform analogous reactions one could expect that maintaining only one set of genes coding for enzymes with broad substrate specificity would be efficient and favorable for energy-limited organisms, such as the sulfate-reducing *D. anilini*. However, we revealed that for the initial degradation steps only the carboxylase subunit Ppcβ2 (02059) from the phenol-degrading gene cluster is used for both aniline and phenol degradation. Possibly, we observe in *D. anilini* a snapshot in the evolution of its genome to become efficient in degradation of aromatics, or alternatively, the struggle of *D. anilini* to survive with a very challenging substrate, aniline, that requires the recruitment of all available and suitable proteins.

The further enzymes involved in the initial steps in aniline degradation comprise a putative 4-aminobenzoyl-CoA ligase (annotated as Acyl-CoA synthetase) and a putative 4-aminobenzoyl-CoA reductase (gene locus tags 03870 and 03876, respectively). Both proteins were highly induced with both aniline and 4-aminobenzoate as sole carbon source, and the encoding genes are located in the aniline-degrading gene cluster *ani* (Figures 1, 2, 3A). The proteomes of aniline-and 4-aminobenzoate-grown cells did not differ significantly suggesting that the genes in the *ani* gene cluster are co-expressed although not all of them are required for 4-aminobenzoate degradation in *D. anilini*. Such co-expression of physically linked genes occurs frequently in eukaryotes (Kruglyak and Tang, 2000; Boutanaev et al., 2002; Lercher et al., 2003) and genes involved in the same pathway tend to be linked (Lee and Sonnhammer, 2003).

Because of the analogous enzymatic functions of the phenol- and aniline-degrading gene cluster for the initial steps in breakdown of phenol or aniline by an endergonic ATP-dependent phosphate synthase reaction followed by an aromatic carboxylation reaction, we compared the respective genes from both gene clusters as well as with the other available related phenol degradation gene cluster from *T. aromatica* K172 [Schmeling et al., 2004; Xie and Müller, 2018 (Supplementary Figures S11–S13)]. The amino acid sequences of the respective genes for phenol and aniline degradation in *D. anilini* do not exhibit high identity (maximum 36% identity within *D. anilini* and maximum 42% identity between *D. anilini* and *T. aromatica*), suggesting that they are of different origin (Supplementary Figures S11–S13). The investigated proteins also share similarities with the respective proteins in *Geobacter metallireducens* GS-15 (Aklujkar et al., 2009) or *Aromatoleum aromaticum* EbN1 (Rabus et al., 2005), yet also with low identities (Supplementary Figure S15). The recruitment of the essential carboxylase subunit from phenol degradation (Ppcβ2, 02059) for aniline degradation is therefore of great interest and calls for further studies on how the gene of this enzyme is selectively expressed from gene cluster *phe* to catalyze also aniline degradation. Strikingly, together with the carboxylase subunit Ppcβ (03872) from the aniline gene cluster, Ppcβ2 converted only phenylphosphoamidate to 4-aminobenzoate but not the related phenylphosphate to 4-hydroxybenzoate. The phenylphosphoamidate synthases of the aniline degradation gene cluster (Pasαβ) instead converted *in vitro* either aniline or phenol to phenylphosphoamidate or phenylphosphate, respectively. The phenylphosphoamidate synthase exhibited a relaxed substrate specificity, but the subunits are only weakly produced when phenol is supplied as sole carbon source (Figure 3, Supplementary Table S1, Xie and Müller, 2018).

Phenylphosphate synthase α-subunit (02052) contains a conserved His569 that is initially phosphorylated (Narmandakh et al., 2006). Protein alignment revealed that the amino acid sequence of phenylphosphoamidate synthase α-subunit (Pasα) contains His512 that corresponds to His571 of phenylphosphate synthase α-subunit (Ppsα, Supplementary Figure S11) or to the conserved histidine residue His569 of phenylphosphate synthase α-subunit of *Thauera aromatica* K172 (Schmeling et al., 2004, Supplementary Figure S11). Consequently, we suggest that His512 catalyzes the nucleophilic attack of the β-phosphoryl group of ATP that transfers the diphosphate group to histidine. Phosphorylated histidine is formed by release of phosphate from pyrophosphate. Then, the electron pair of the amino group of aniline attacks the phosphate group of the phosphorylated histidine to form phenylphosphoamidate. Lack of the γ-subunit in *D. anilini* may explain the lower turnover rate of aniline in *D. anilini*, compared to phenol in *T. aromatica* (Schmeling et al., 2004).

Phenylphosphoamidate is carboxylated to 4-aminobenzoate by phenylphosphoamidate carboxylase (phenylphosphate carboxylase β-subunits Ppcβ and Ppcβ2) (Figure 1). The phenylphosphoamidate carboxylase is ATP-independent, suggesting a reaction mechanism different from well-established ATP- and biotin-dependent carboxylases (Attwood, 1995; Attwood and Wallace, 2002). Instead, phenylphosphoamidate carboxylase requires Mn^2+^, analogous to UbiD (Leppik et al., 1976). *T. aromatica* requires two UbiD-like subunits for the phenylphosphate carboxylase to carboxylate phenylphosphate (Breinig et al., 2000; Schühle and Fuchs, 2004). Similar to the phenylphosphate carboxylase complex in *T. aromatica*, the phenylphosphoamidate carboxylase complex in *D. anilini* also contains two UbiD-like subunits (Ppcβ, 03872 and Ppcβ2, 02059) sharing 52% amino acid identities (Supplementary Figure S13), but without the γ- or δ-subunit.

UbiD carboxylases usually require prenylated FMN (prFMN) that is produced by UbiX (Marshall et al., 2017). However, for the carboxylation of phenylphosphoamidate, FMN is sufficient to catalyze the formation of 4-aminobenzoate by the two UbiD-like subunits of phenylphosphoamidate carboxylase (Ppcβ, 03872 and Ppcβ2, 02059). BLAST-search of the amino acid sequence of *ubiX* of *Escherichia coli* K-12, MG1655 revealed, that a *ubiX*-like gene with 52% amino acid identity (4-hydroxy-3-polyprenylbenzoate-decarboxylase) is located in gene cluster *phe* of *D. anilini* (locus tag 02056, Figure 1). However, the protein of the *ubiX*-like gene can only be found in the proteome during growth with phenol (Supplementary Table S1). Future research will reveal, whether co-expression of *ubiX* with the other genes of aniline degradation in mutant strains of *D. anilini* enhances the kinetic parameters of the aniline-degrading enzymes.

**FIGURE 8:**
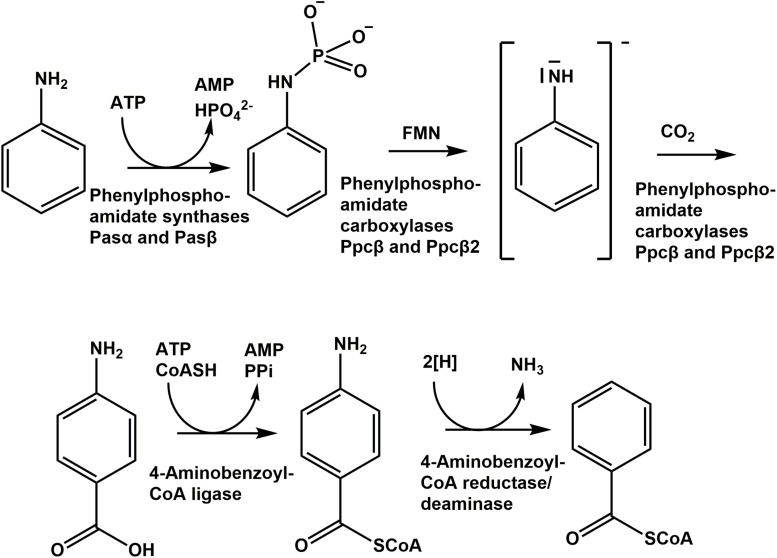
Proposed pathway of anaerobic aniline degradation by *D. anilini*.

Likely, the phenylphosphoamidate carboxylase of *D. anilini* generates an activated anilinide (-like) intermediate in analogy to the suggested mechanism of phenol degradation via a phenolate intermediate (Figure 8; Schmeling et al., 2004). This anilinide intermediate then can attack carbon dioxide to form 4-aminobenzoate. The phosphate-like moiety of the phenylphosphoamidate is most likely required to facilitate the reduction of the amino group of aniline, possibly by FMN (Figure 8), constituting a good leaving group and thus promoting the anilinide-like intermediate formation.

Our heterologous expression of the carboxylase subunits from *D. anilini* should set the ground for future mechanistic studies of the remarkable aromatic carboxylation reaction of phenylphosphoamidate (as well as phenylphosphate). Moreover, degradation of either aniline or phenol forces *D. anilini* to invest a high amount of energy into their initial activation to phenylphosphoamidate or phenylphosphate, respectively. Future experiments are needed to reveal how *D. anilini* can still maintain an overall positive ATP balance and survive with aniline as sole carbon source.

## Data Availability Statement

All data presented is included in the article. Proteomics data was added in the form of an Excel-file in the (Supplementary Table S1). Further raw data supporting the conclusions of this article will be made available upon request and without undue reservation to any qualified researcher.

## Author Contributions

XX performed the experiments designed by XX, NM, BS, and DS. Phenylphosphoamidate was synthesized by TH. LC-MS measurements were conducted by DS. Statistical analysis of Proteomics data was performed by NM. All authors analyzed the data, contributed to writing the manuscript and approved the final version of the manuscript.

## Conflict of Interest

The authors declare that the research was conducted in the absence of any commercial or financial relationships that could be construed as a potential conflict of interest.

## Footnotes

1 https://img.jgi.doe.gov/

## Supplementary Material

FIGURE S1 ^1^H-NMR-spectrum of tribenzyl phosphite (400 MHz, DMSO).

FIGURE S2 ^31^P-NMR-spectrum of tribenzyl phosphite (162 MHz, DMSO).

FIGURE S3 ^1^H-NMR-spectrum of dibenzyl *N*-phenylphosphoramidate (400 MHz, CDCl_3_).

FIGURE S4 ^31^P-NMR -spectrum of dibenzyl *N*-phenylphosphoramidate (162 MHz, CDCl_3_).

FIGURE S5 ^13^C-NMR -spectrum of dibenzyl *N*-phenylphosphoramidate (101 MHz, CDCl_3_).

FIGURE S6 ^1^H-NMR-spectrum of triethylammonium *N*-phenylphosphoramidate (400 MHz, CD_3_OD).

FIGURE S7 ^13^C NMR-spectrum of triethylammonium *N*-phenylphosphoramidate (101 MHz, CD_3_OD).

FIGURE S8 ^31^P-NMR-spectrum of triethylammonium *N*-phenyl- phosphoramidate (162 MHz, CD_3_OD).

FIGURE S9 Total proteome analysis of cell-free extracts from the cells of *D. anilini* grown on phenol (purple bars), benzoate (green bars), aniline (red bars) and 4-aminobenzoate (blue bars), as sole carbon source, respectively. Protein abundances are represented as area values of the MS-signals of peptides identified with the Proteome Discoverer software. The logarithm to the base 10 of mean values and standard deviations of the protein abundances (area) in cell-lysates from three independent cultures under the respective growth condition are shown (AU: arbitrary units). *Z*-scores are shown as a standardized representation of the data. Positive *Z*-scores represent values above the average, negative *Z*-scores represent values below the average. The highest observed *Z*-score was 27.1 (H567DRAFT_02821 dissimilatory adenylylsulfate reductase alpha subunit precursor) and the lowest observed *Z*-score was -0.23 (H567DRAFT_04108 hemerythrin-like domain-containing protein). Abundances of the enzymes encoded by gene cluster *phe* (locus tag 02049-02059) are presented.

FIGURE S10 Volcano plot of the total proteomics data of three independent cultures of aniline- and phenol-grown cells. The negative logarithm to base 10 of the *p*-values of two-sided, paired *t*-tests are plotted against the logarithm to the base 2 of the ratio of the mean area values of aniline- versus phenol-grown cells. The red line represents the weak significance threshold (*p* < 0.1) and the yellow line represents the strong significance threshold (*p* < 0.05).

FIGURE S11 Alignment of amino acid sequences of phenylphosphoamidate synthase α subunit (Pasα), phenylphosphate synthase α-subunit (Ppsα) in *D. anilini* and phenylphosphate synthase α-subunit in *Thauera aromatica* K172 (IMG-locus tag Ga0309627_112830) Numbers of conserved histidine residues are indicated in blue. Green bars indicate 100% mean pairwise identity, green-brown bars indicate at least 30% and lower than 100% identity.

FIGURE S12 Alignment of amino acid sequences of phenylphosphoamidate synthase β-subunit (Pasβ), phenylphosphate synthase β-subunit (Ppsβ) in *D. anilini* and phenylphosphate synthase β-subunit in *Thauera aromatica* K172 (IMG-locus tag Ga0309627_112831). Green bars indicate 100% mean pairwise identity, green-brown bars indicate at least 30% and lower than 100% identity.

FIGURE S13 Alignment of amino acid sequences of phenylphosphoamidate carboxylase β-subunit (Ppcβ), phenylphosphate carboxylase β-subunit (Ppcβ2) in *D. anilini* and phenylphosphate carboxylase β-subunit in *Thauera aromatica* K172 (IMG-locus tag Ga0309627_112833) Green bars indicate 100% mean pairwise identity, green-brown bars indicate at least 30% and lower than 100% identity.

FIGURE S14 Non enzymatic breakdown of phenylphosphoamidate to aniline over time at different pHs. The amount of phenylphosphoamidate and aniline are represented by the peak area measured by HPLC and photodiode array detection. Phenylphosphosamidate at pH 7.0 (solid square); aniline at pH 7.0 (empty square); phenylphosphoamidate at pH 8.0 (solid circle); aniline at pH 8.0 (empty circle).

FIGURE S15 Phylogenetic distance trees of the amino acid sequences of Pasα **(A)**, Pasβ **(B)**, and Ppcβ **(C)**. The bars represent 0.5 or 1 amino acid substitutions.
