# Supplementary figures and images for: Desulfatiglans anilini Initiates Degradation of Aniline With the Production of Phenylphosphoamidate and 4-Aminobenzoate as Intermediates Through Synthases and Carboxylases From Different Gene Clusters
Source: Front Microbiol. 2020 Sep 4;11:2064. doi: 10.3389/fmicb.2020.02064 (PMC7500099; doi:10.3389/fmicb.2020.02064)

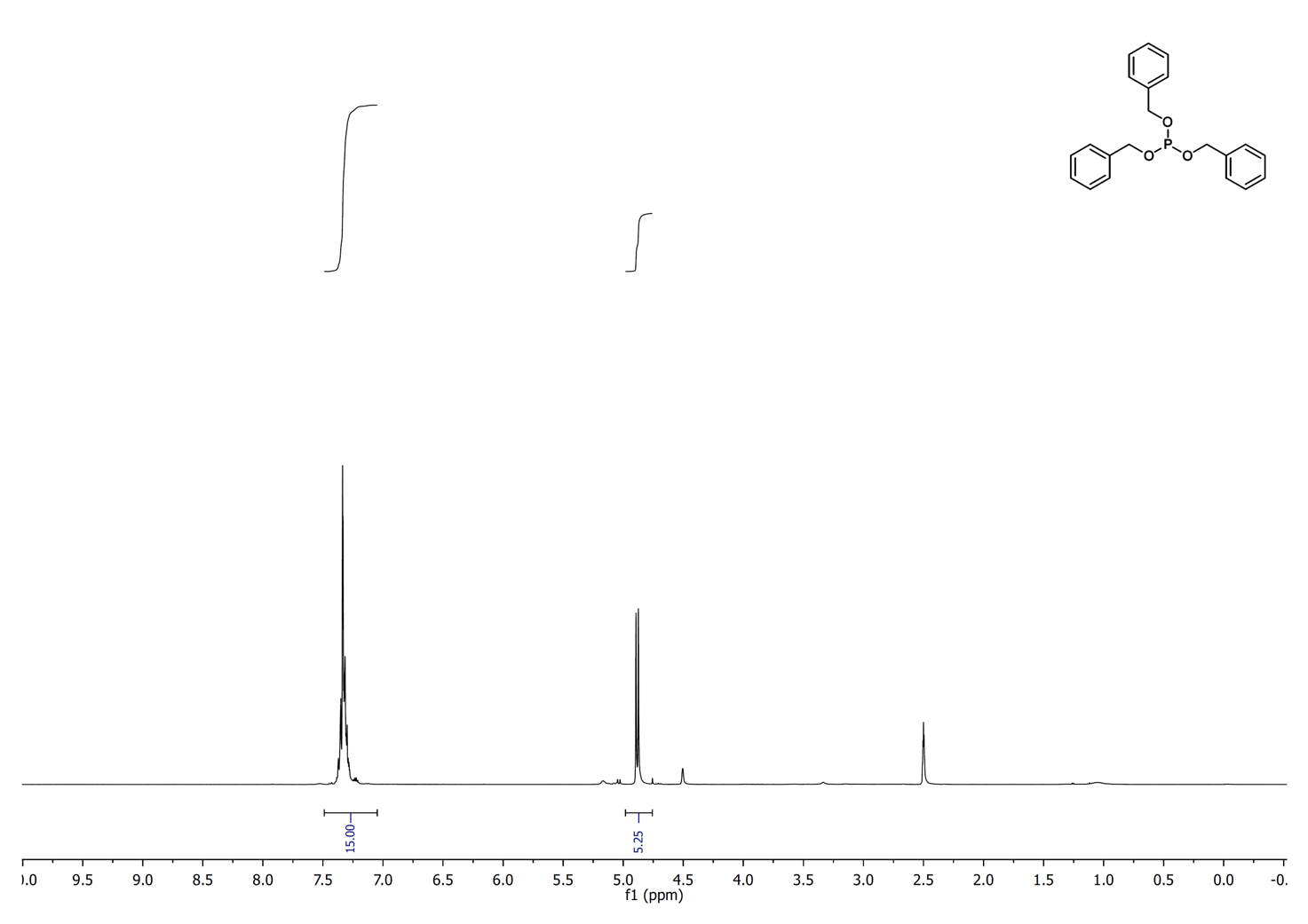

Supplement: FIGURE S1 — 1H-NMR-spectrum of tribenzyl phosphite (400 MHz, DMSO). [file Image_1.TIF]

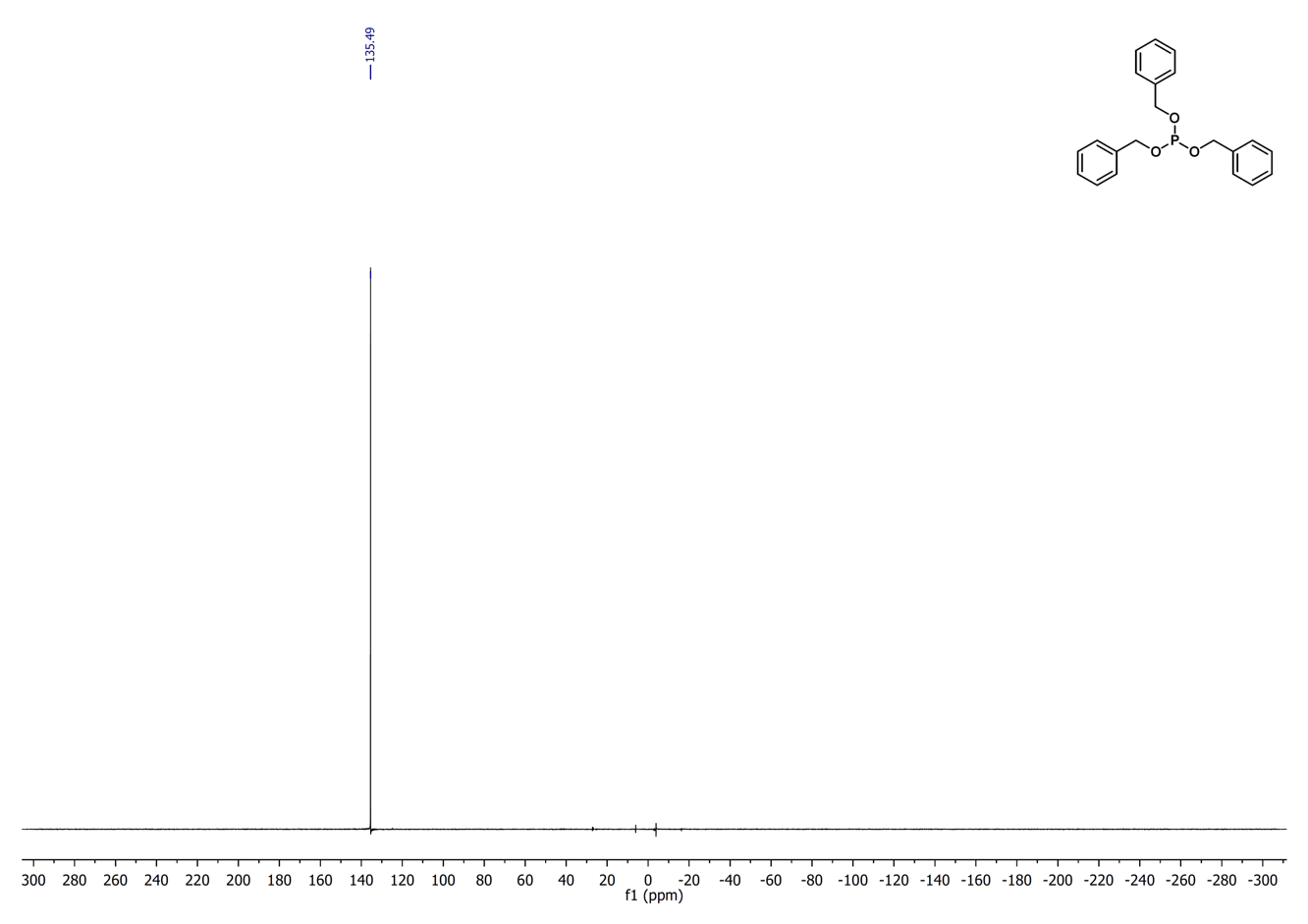

Supplement: FIGURE S2 — 31P-NMR-spectrum of tribenzyl phosphite (162 MHz, DMSO). [file Image_2.TIF]

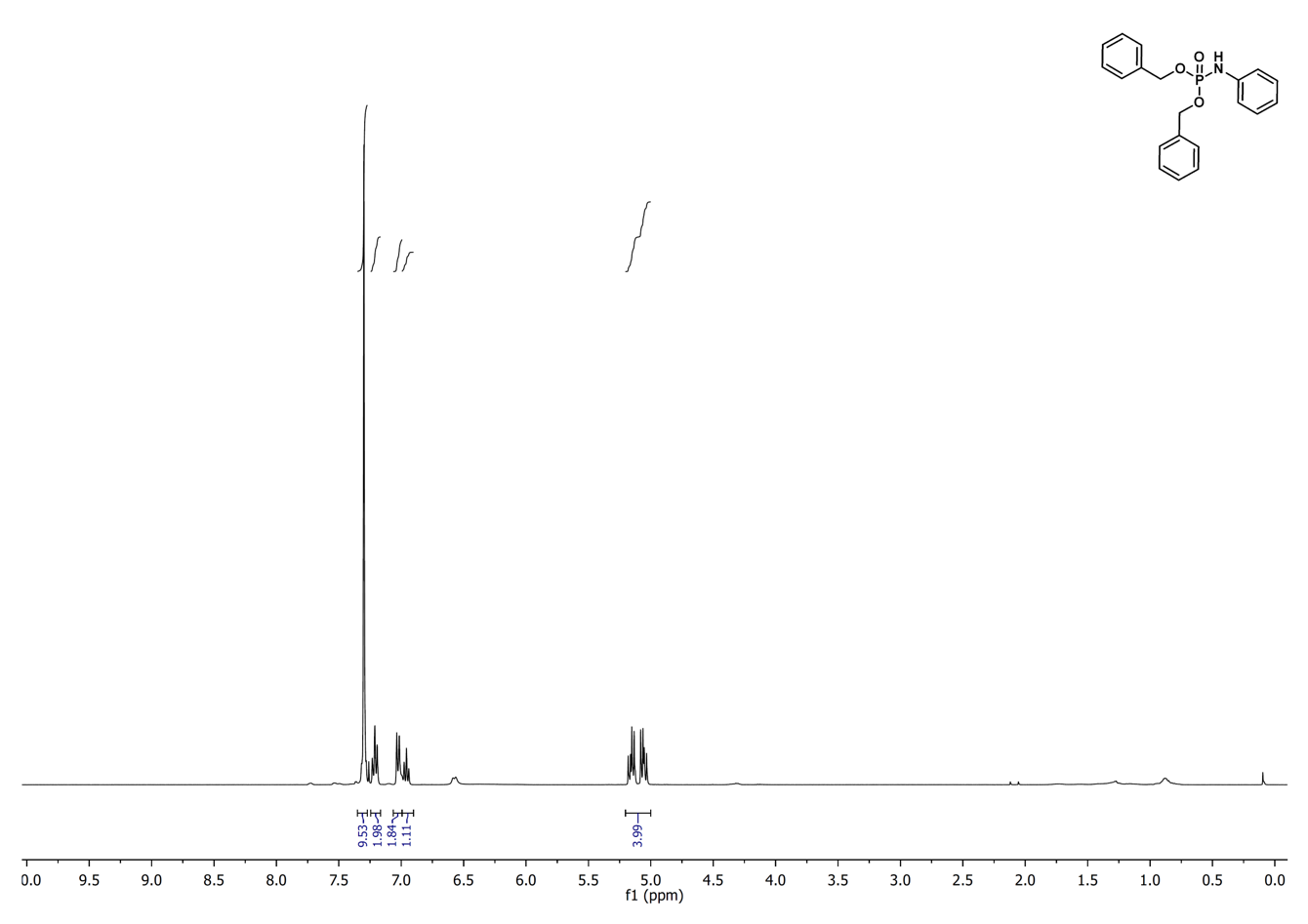

Supplement: FIGURE S3 — 1H-NMR-spectrum of dibenzyl N-phenylphosphoramidate (400 MHz, CDCl3). [file Image_3.TIF]

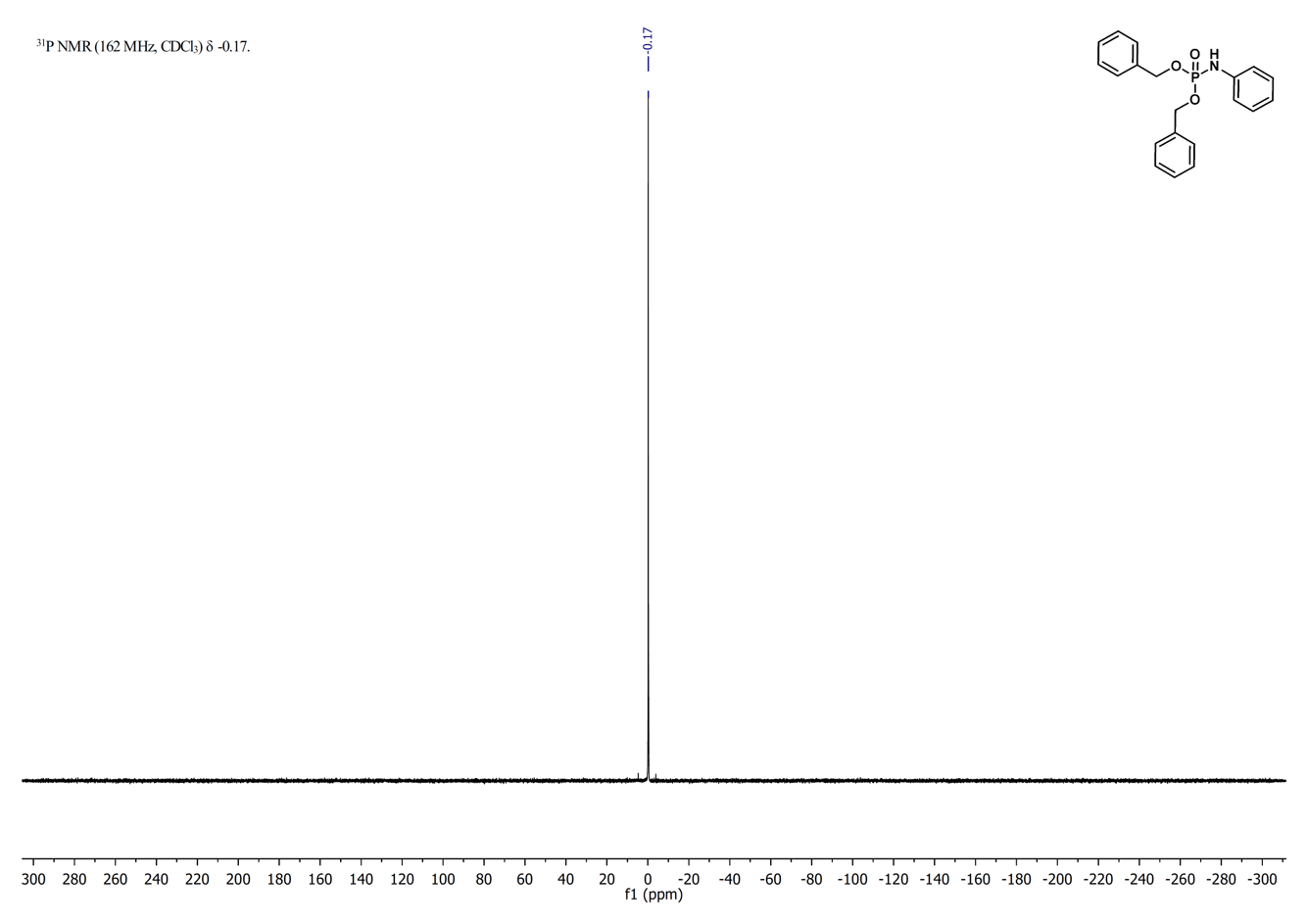

Supplement: FIGURE S4 — 31P-NMR -spectrum of dibenzyl N-phenylphosphoramidate (162 MHz, CDCl3). [file Image_4.TIF]

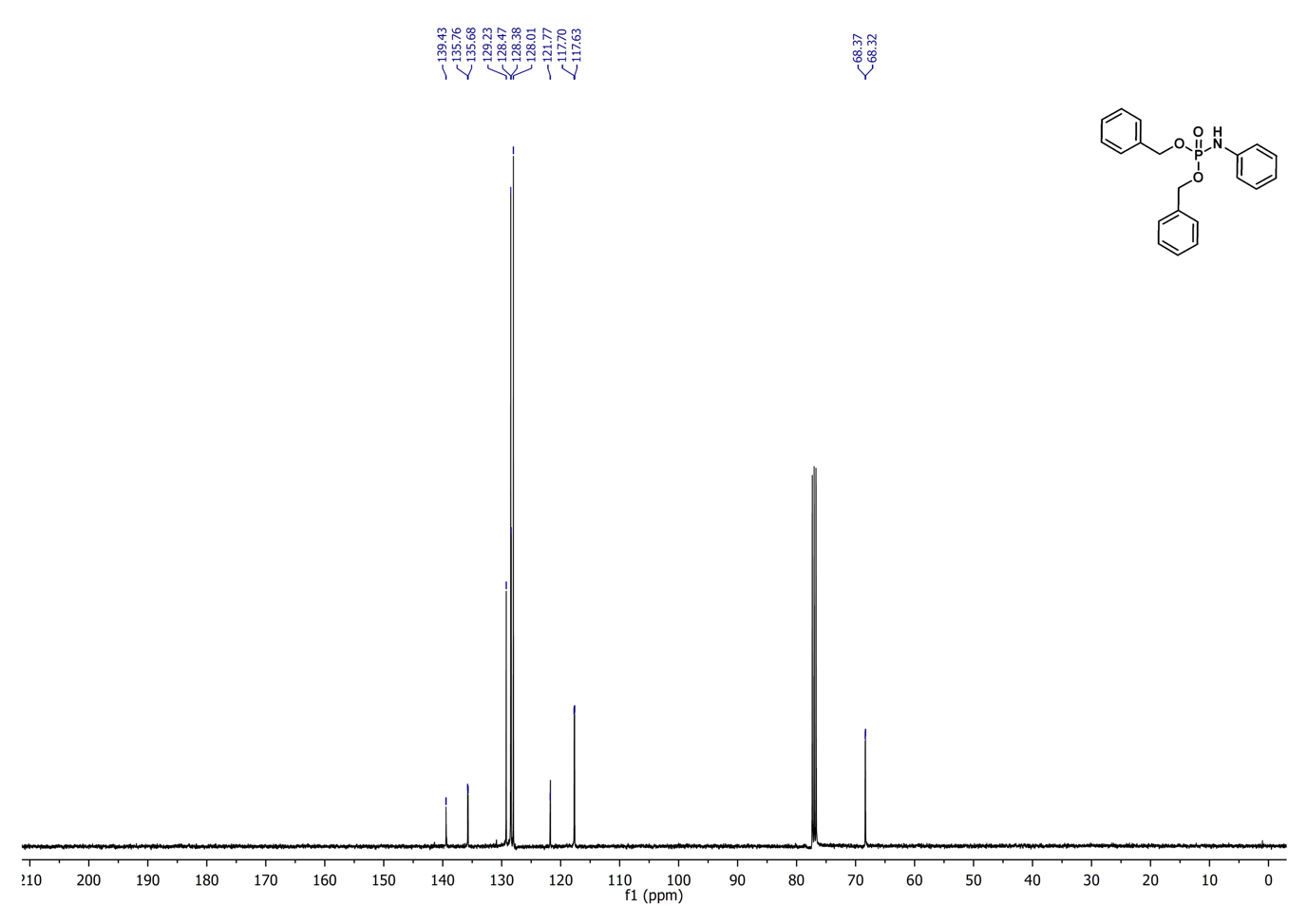

Supplement: FIGURE S5 — 13C-NMR -spectrum of dibenzyl N-phenylphosphoramidate (101 MHz, CDCl3). [file Image_5.TIF]

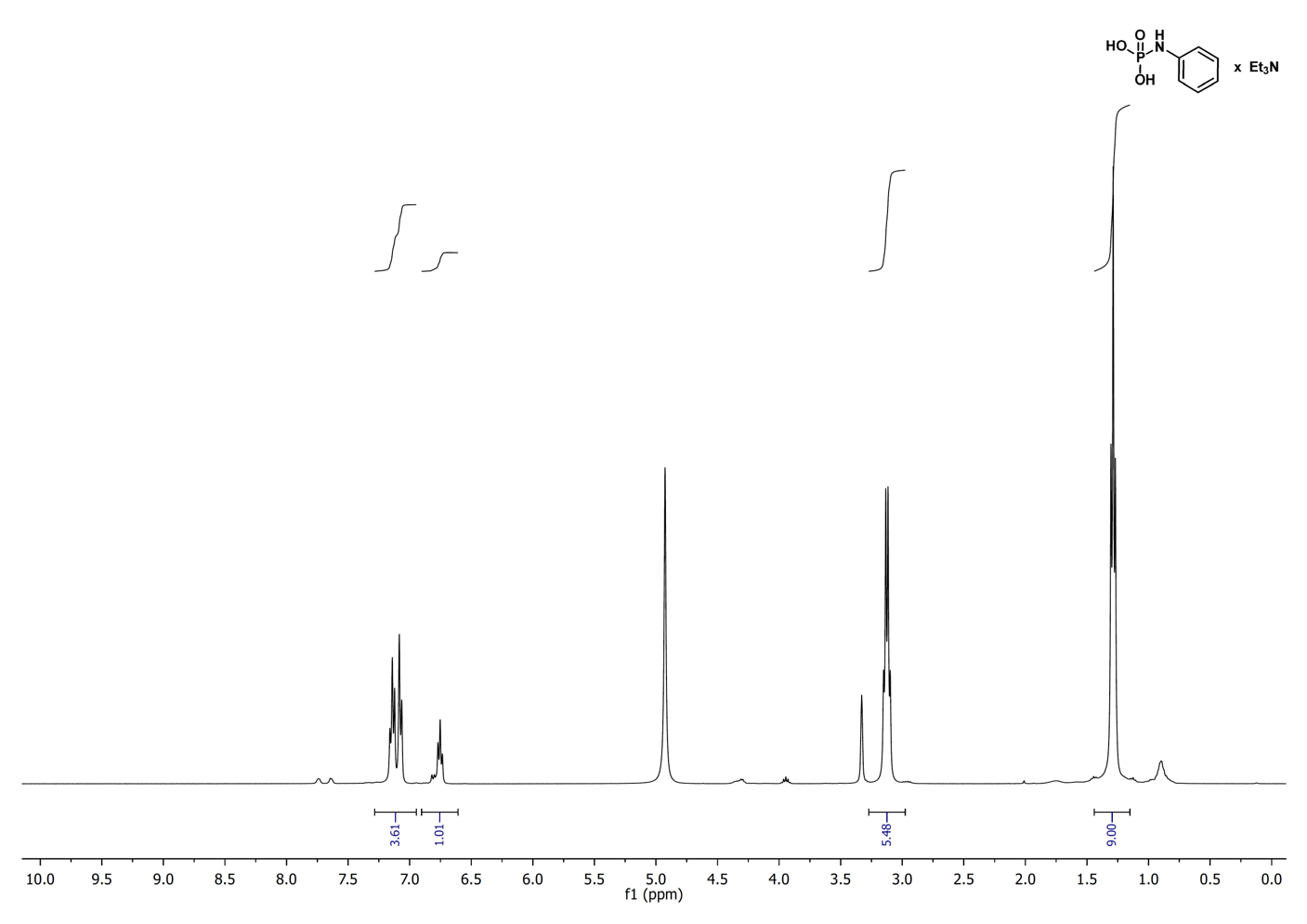

Supplement: FIGURE S6 — 1H-NMR-spectrum of triethylammonium N-phenylphosphoramidate (400 MHz, CD3OD). [file Image_6.TIF]

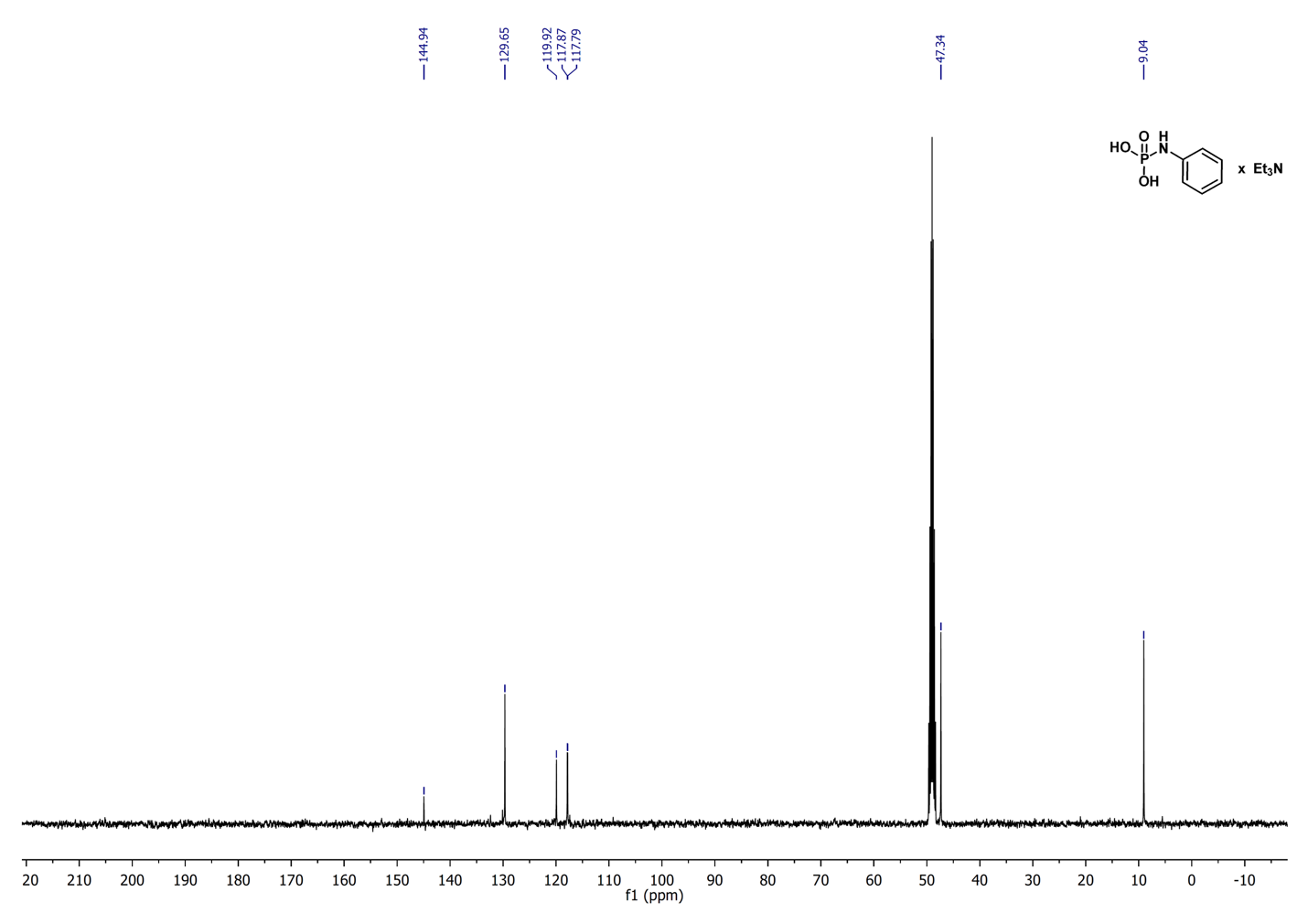

Supplement: FIGURE S7 — 13C NMR-spectrum of triethylammonium N-phenylphosphoramidate (101 MHz, CD3OD). [file Image_7.TIF]

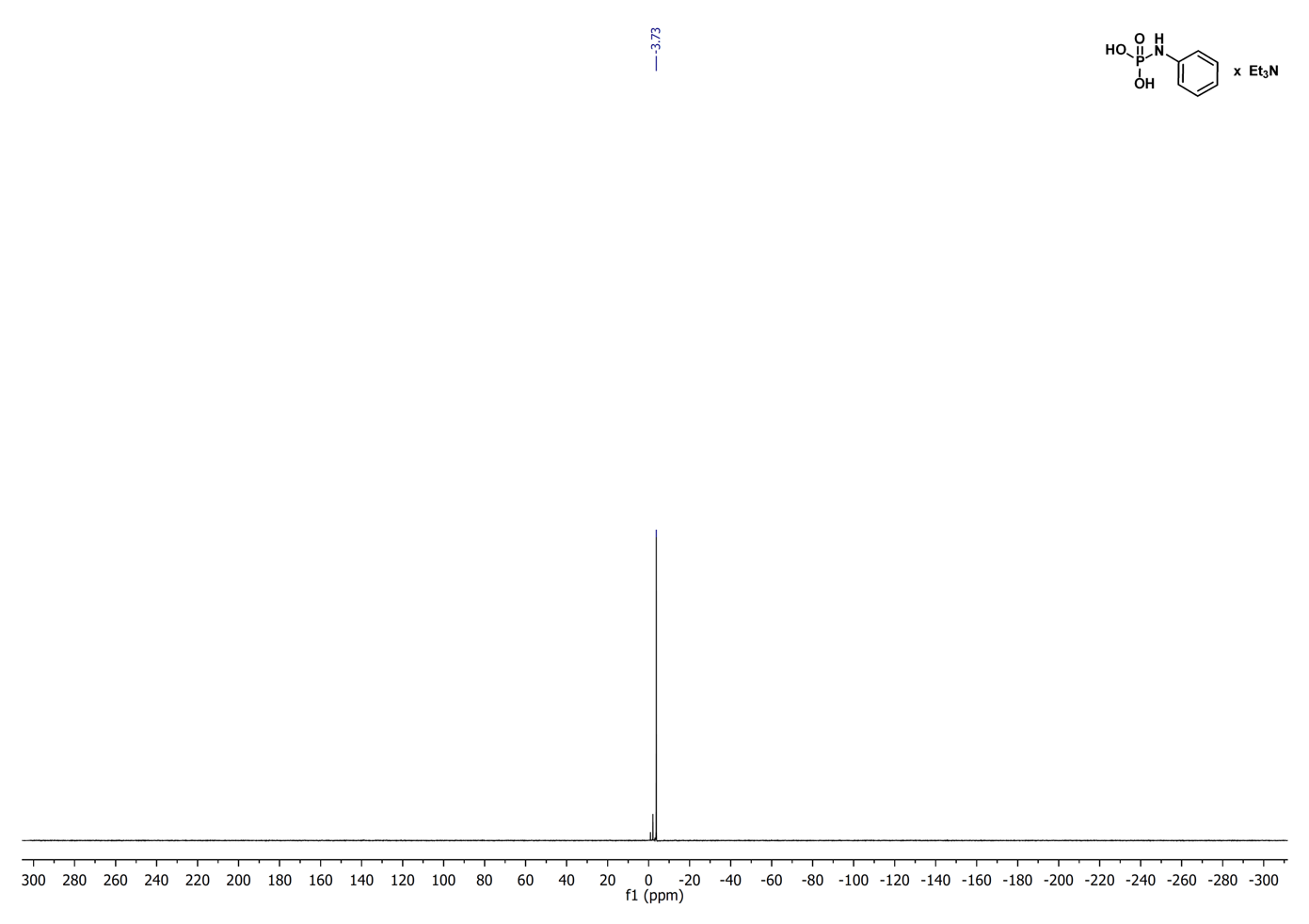

Supplement: FIGURE S8 — 31P-NMR-spectrum of triethylammonium N-phenyl- phosphoramidate (162 MHz, CD3OD). [file Image_8.TIF]

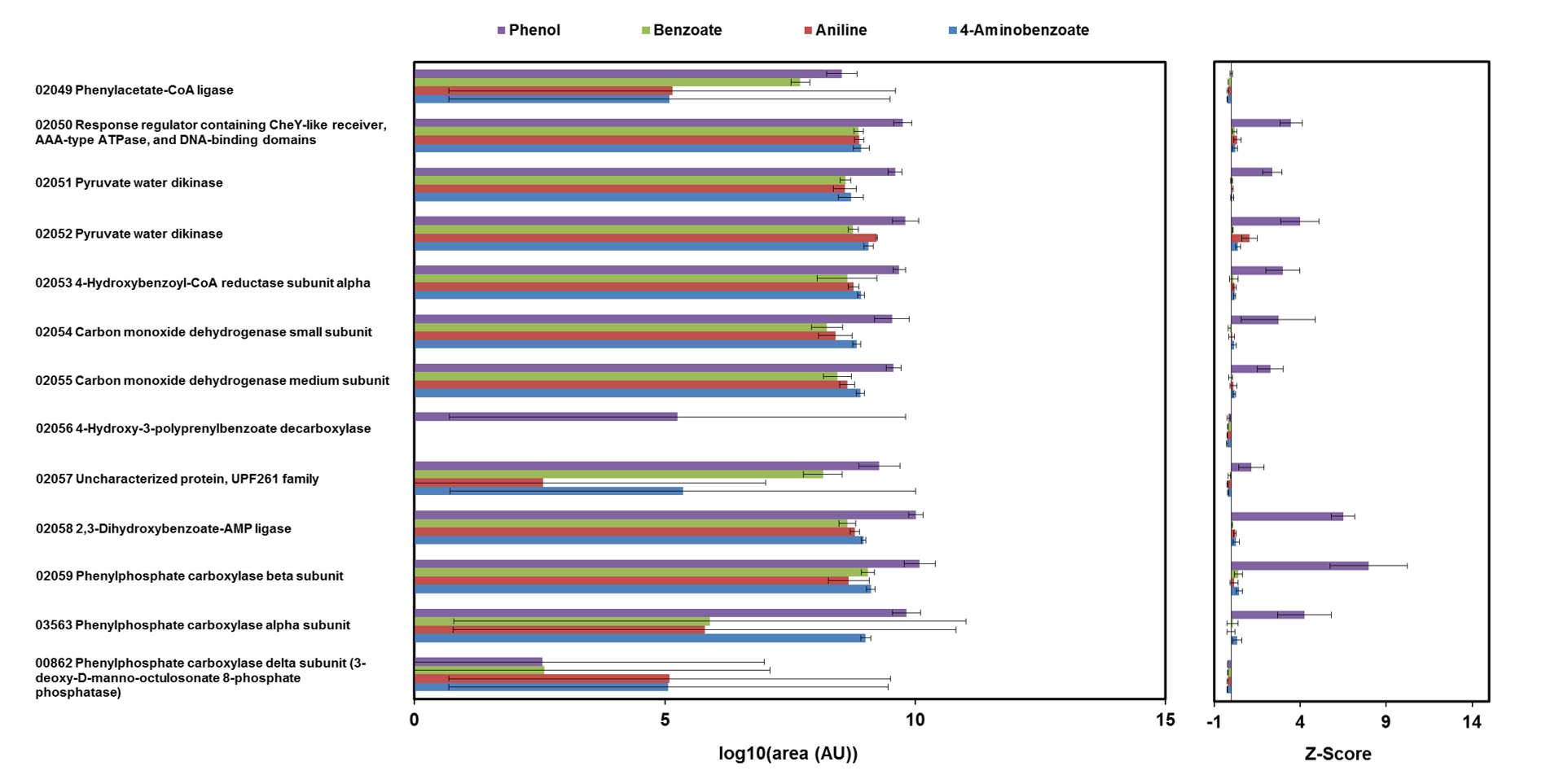

Supplement: FIGURE S9 — Total proteome analysis of cell-free extracts from the cells of D. anilini grown on phenol (purple bars), benzoate (green bars), aniline (red bars) and 4-aminobenzoate (blue bars), as sole carbon source, respectively. Protein abundances are represented as area values of the MS-signals of peptides identified with the Proteome Discoverer software. The logarithm to the base 10 of mean values and standard deviations of the protein abundances (area) in cell-lysates from three independent cultures under the respective growth condition are shown (AU: arbitrary units). Z-scores are shown as a standardized representation of the data. Positive Z-scores represent values above the average, negative Z-scores represent values below the average. The highest observed Z-score was 27.1 (H567DRAFT_02821 dissimilatory adenylylsulfate reductase alpha subunit precursor) and the lowest observed Z-score was -0.23 (H567DRAFT_04108 hemerythrin-like domain-containing protein). Abundances of the enzymes encoded by gene cluster phe (locus tag 02049-02059) are presented. [file Image_9.TIF]

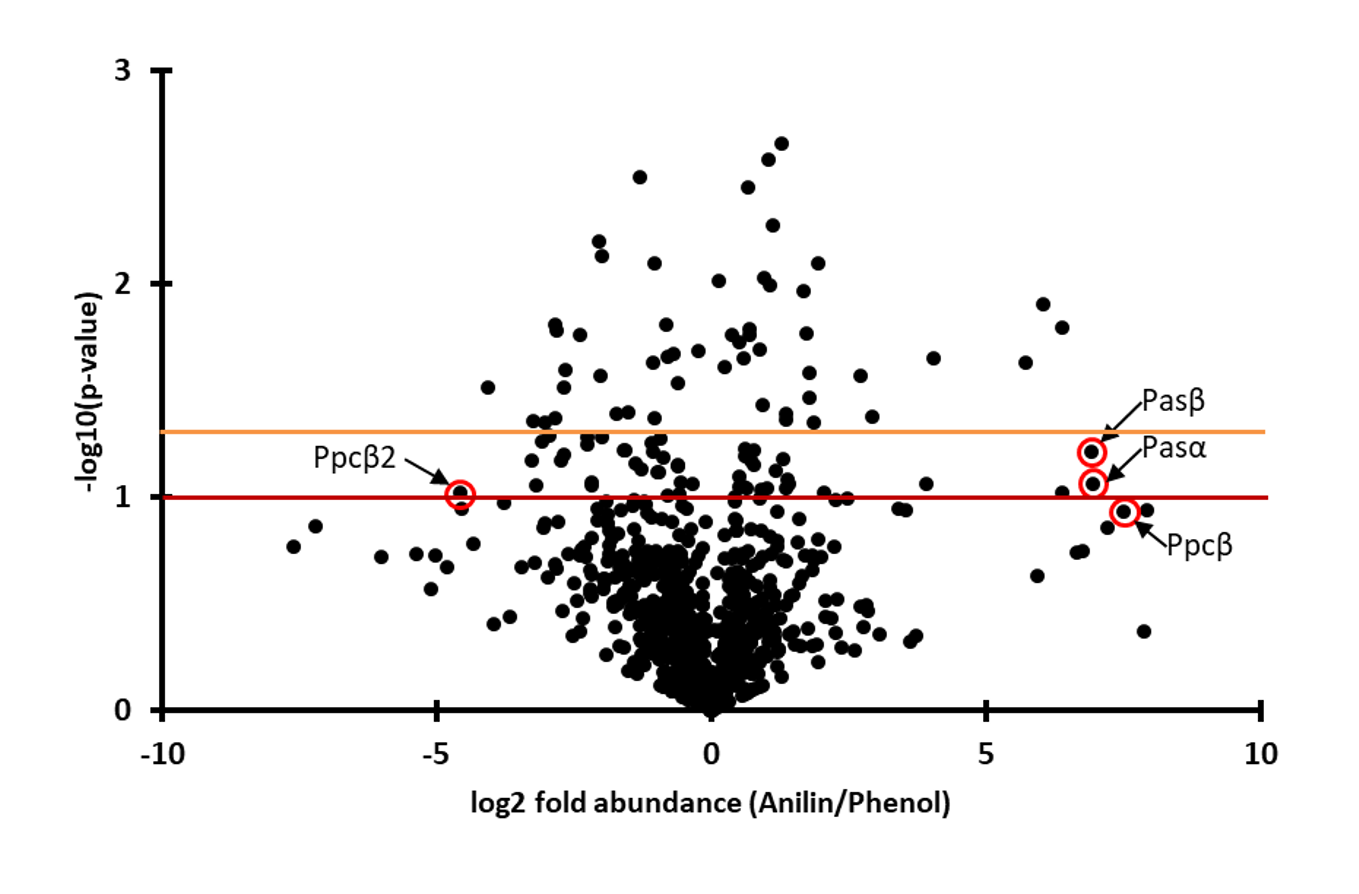

Supplement: FIGURE S10 — Volcano plot of the total proteomics data of three independent cultures of aniline- and phenol-grown cells. The negative logarithm to base 10 of the p-values of two-sided, paired t-tests are plotted against the logarithm to the base 2 of the ratio of the mean area values of aniline- versus phenol-grown cells. The red line represents the weak significance threshold (p < 0.1) and the yellow line represents the strong significance threshold (p < 0.05). [file Image_10.TIF]

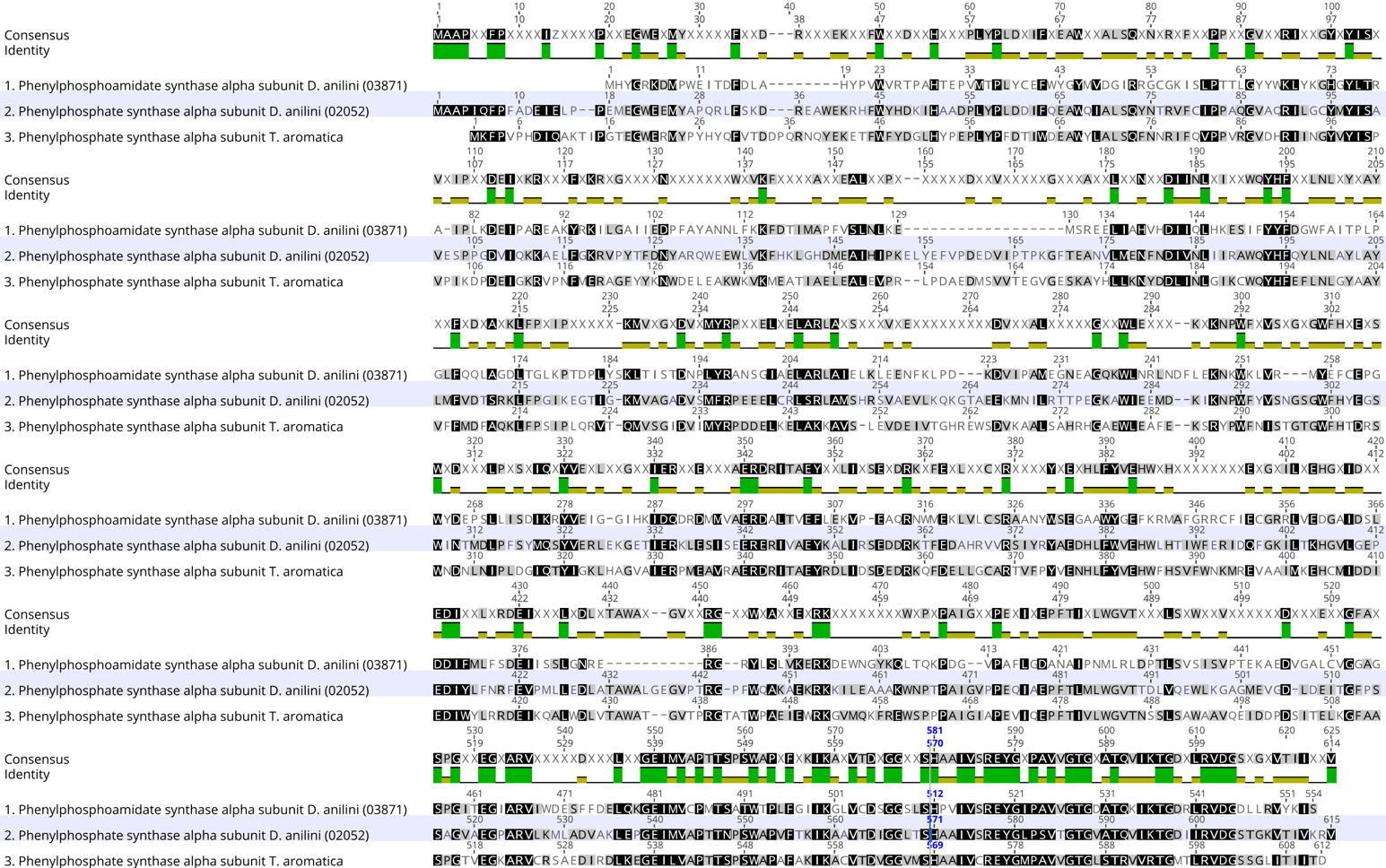

Supplement: FIGURE S11 — Alignment of amino acid sequences of phenylphosphoamidate synthase α subunit (Pasα), phenylphosphate synthase α-subunit (Ppsα) in D. anilini and phenylphosphate synthase α-subunit in Thauera aromatica K172 (IMG-locus tag Ga0309627_112830) Numbers of conserved histidine residues are indicated in blue. Green bars indicate 100% mean pairwise identity, green-brown bars indicate at least 30% and lower than 100% identity. [file Image_11.TIF]

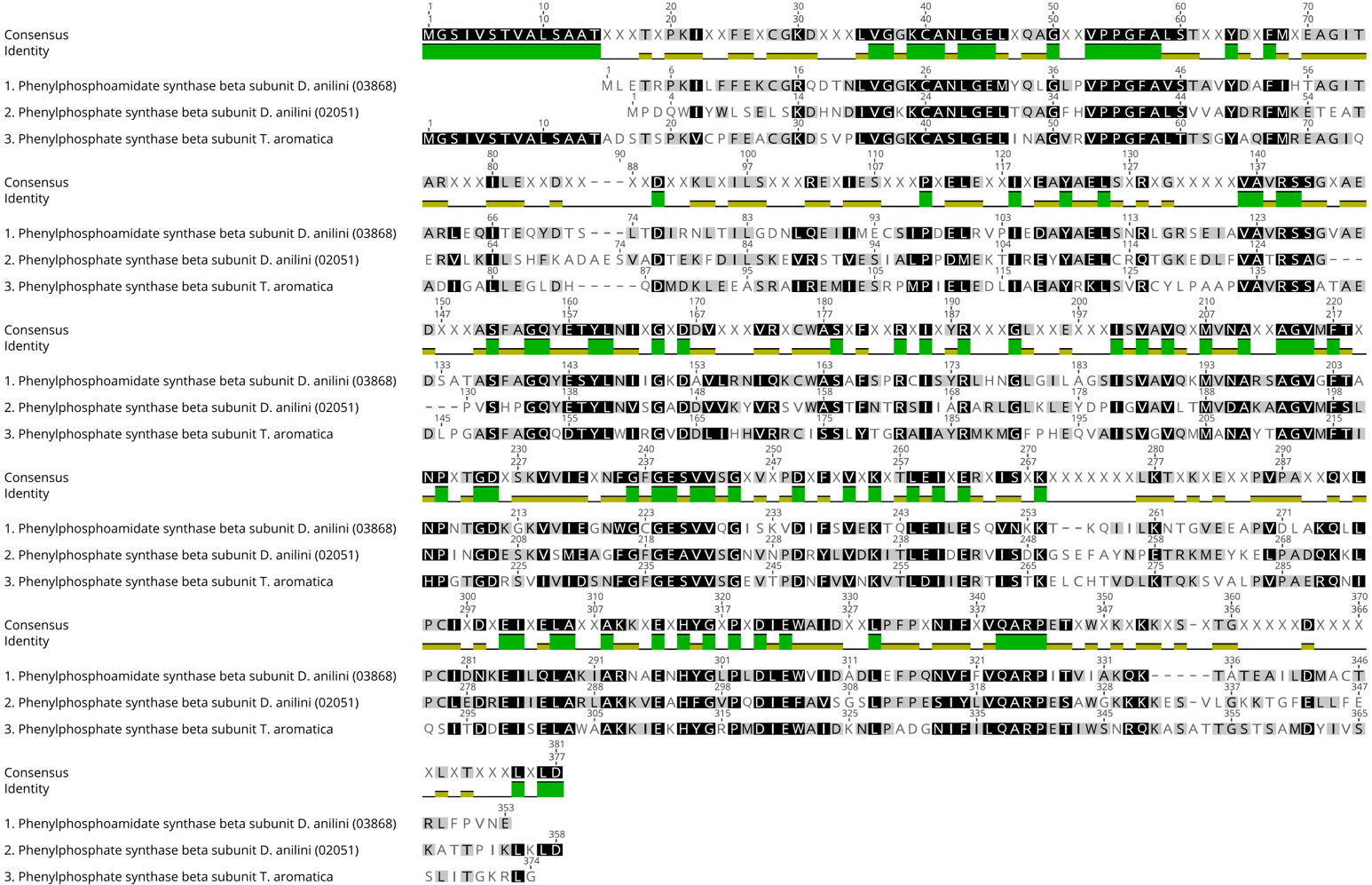

Supplement: FIGURE S12 — Alignment of amino acid sequences of phenylphosphoamidate synthase β-subunit (Pasβ), phenylphosphate synthase β-subunit (Ppsβ) in D. anilini and phenylphosphate synthase β-subunit in Thauera aromatica K172 (IMG-locus tag Ga0309627_112831). Green bars indicate 100% mean pairwise identity, green-brown bars indicate at least 30% and lower than 100% identity. [file Image_12.TIF]

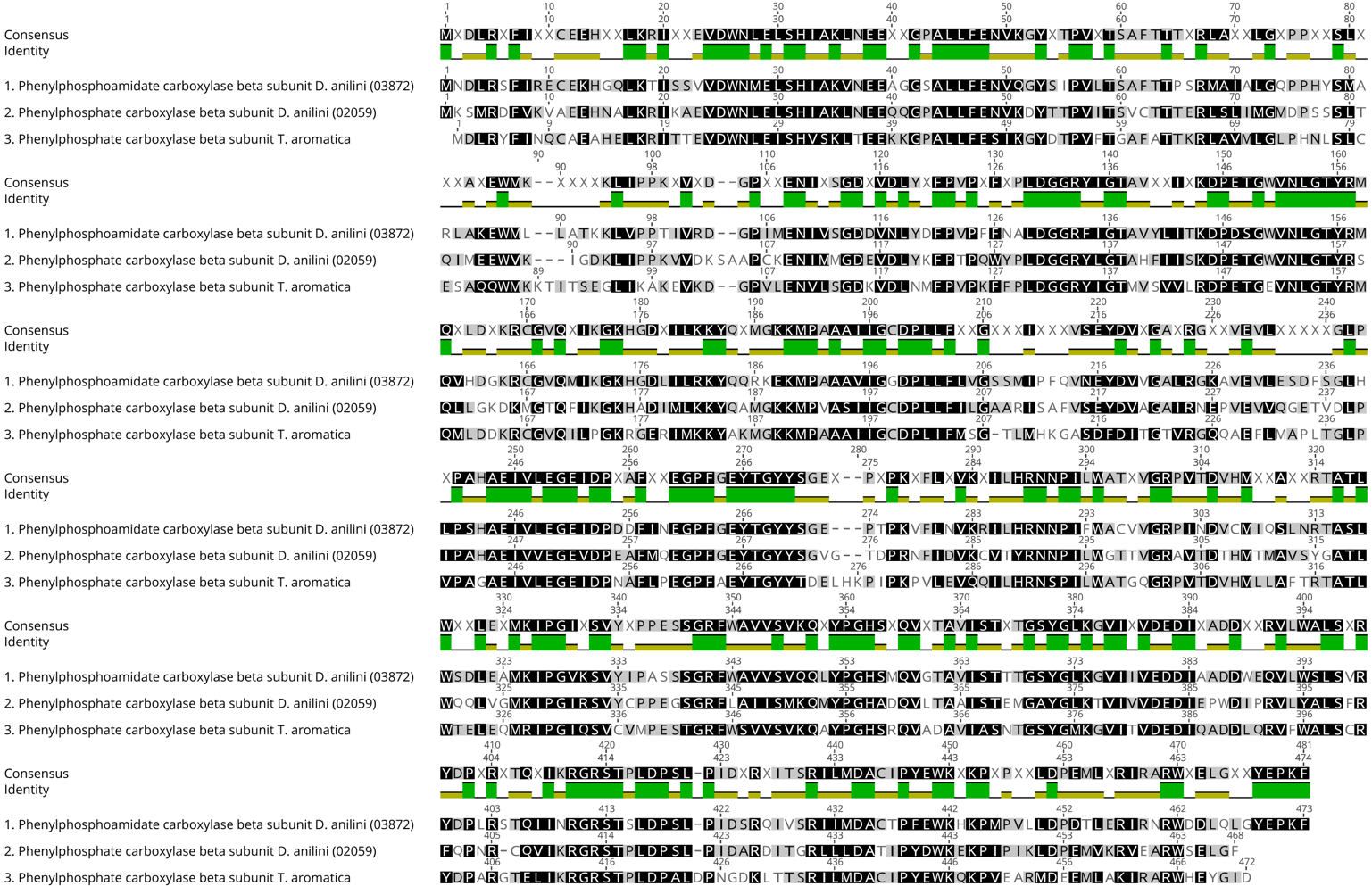

Supplement: FIGURE S13 — Alignment of amino acid sequences of phenylphosphoamidate carboxylase β-subunit (Ppcβ), phenylphosphate carboxylase β-subunit (Ppcβ2) in D. anilini and phenylphosphate carboxylase β-subunit in Thauera aromatica K172 (IMG-locus tag Ga0309627_112833) Green bars indicate 100% mean pairwise identity, green-brown bars indicate at least 30% and lower than 100% identity. [file Image_13.TIF]

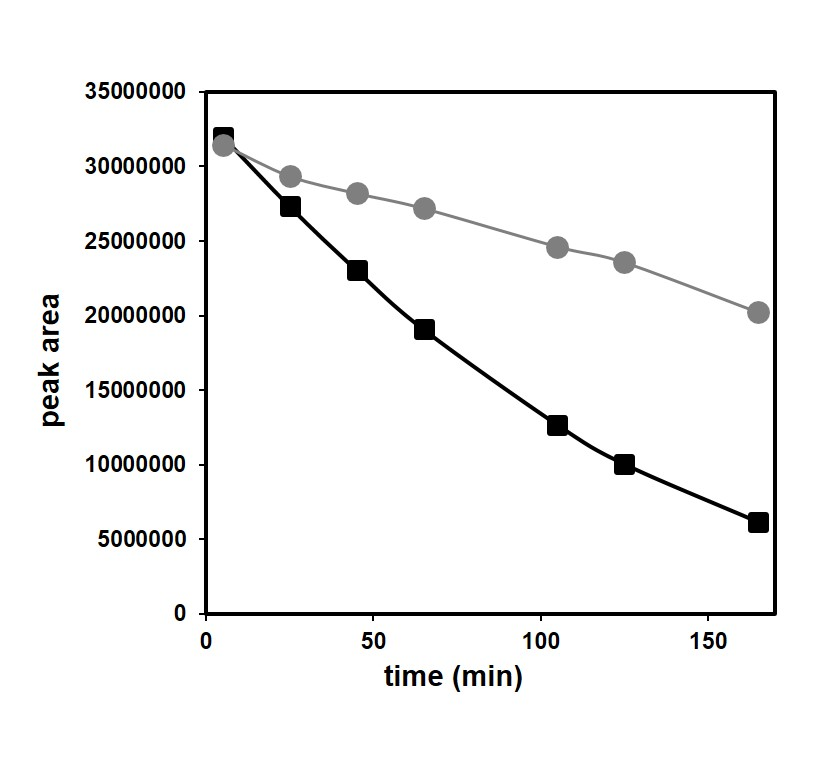

Supplement: FIGURE S14 — Non enzymatic breakdown of phenylphosphoamidate to aniline over time at different pHs. The amount of phenylphosphoamidate and aniline are represented by the peak area measured by HPLC and photodiode array detection. Phenylphosphosamidate at pH 7.0 (solid square); aniline at pH 7.0 (empty square); phenylphosphoamidate at pH 8.0 (solid circle); aniline at pH 8.0 (empty circle). [file Image_14.TIF]

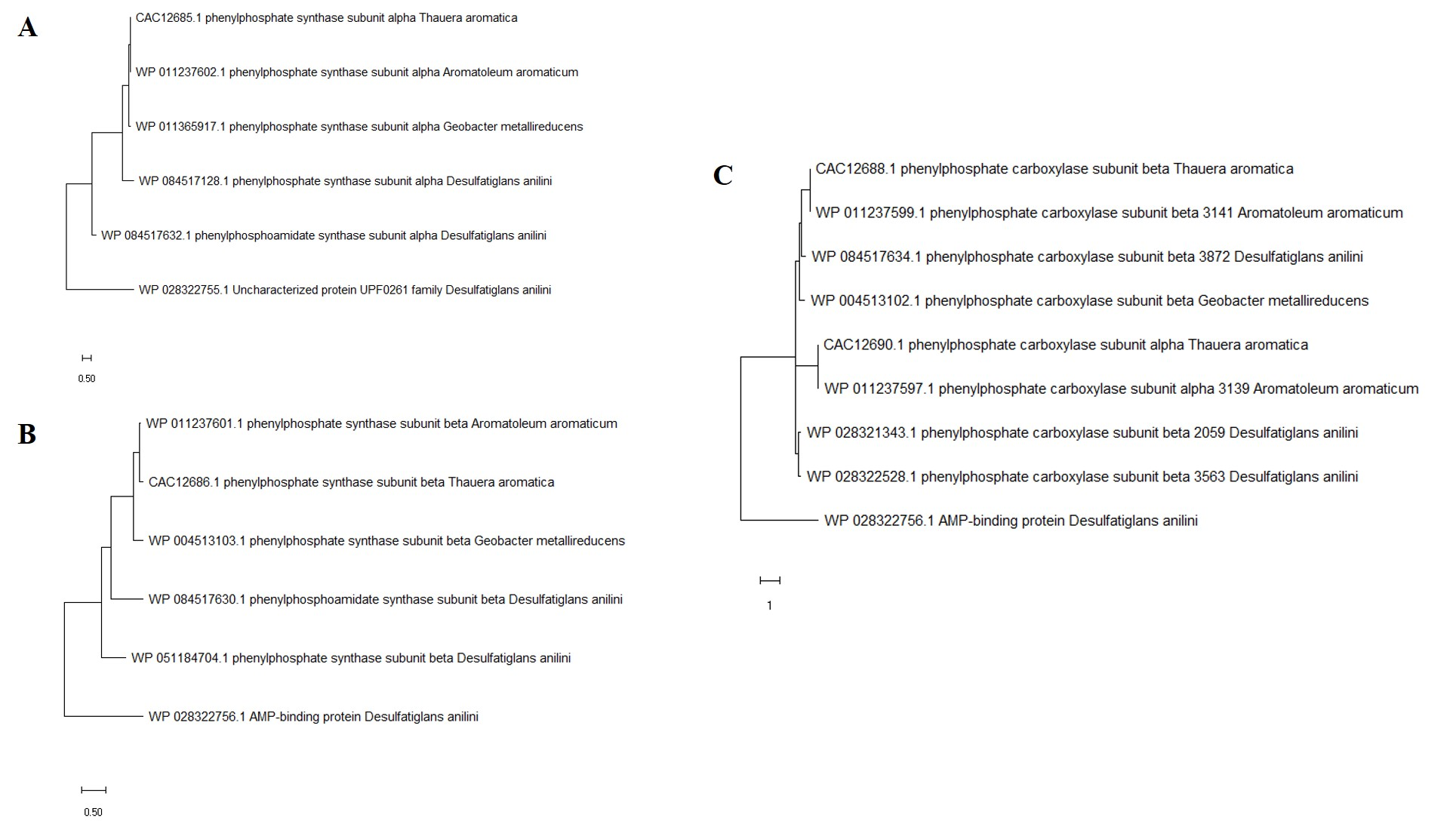

Supplement: FIGURE S15 — Phylogenetic distance trees of the amino acid sequences of Pasα (A), Pasβ (B), and Ppcβ (C). The bars represent 0.5 or 1 amino acid substitutions. [file Image_15.TIFF]
